# The co-creation, initial piloting, and protocol for a cluster randomised controlled trial of a coach-led positive body image intervention for girls in sport

**DOI:** 10.1186/s12889-023-16360-w

**Published:** 2023-07-31

**Authors:** E. L. Matheson, J. Schneider, A. Tinoco, C. Gentili, H. Silva-Breen, N. M. LaVoi, P. White, P. C. Diedrichs

**Affiliations:** 1 Centre for Appearance Research, School of Social Sciences, College of Health, Science and Society, University of the West of England, Coldharbour Lane, Bristol, BS16 1QY UK; 2 Department of Sport, Exercise, and Performance Psychology, West Virginia University, Morgantown, WV 26506 USA; 3 Tucker Center for Research on Girls & Women in Sport, University of Minnesota, 1900 University Avenue SE, Minneapolis, MN 55455 USA

**Keywords:** Adolescent, Girls, Body image, Sports participation, Intervention development, Cluster randomised controlled trial, Study protocol

## Abstract

**Background:**

Globally, girls disengage from sports at an earlier age and higher rate than boys. This is, in part, due to the unique body image challenges that girls face, relative to their male peers. Existing intervention efforts that aim to reduce girls’ negative body image and movement experiences have proven marginally effective, if not ineffective. This paper outlines the co-creation, initial piloting and protocol for a cluster randomised controlled trial of Body Confident Athletes (BCA); an in-person, coach-led intervention that aims to foster positive body image and sports enjoyment among girls.

**Methods:**

Following co-creation and an initial pilot, a two-armed cluster randomised controlled trial will assess the immediate (post-intervention) and short-term (1-month and 3-month follow-up) impact of BCA on girls’ (*N* = 1,036; 11–17 years old) body image, sports enjoyment, and affect. Sport organisations will be randomly allocated (1:1) into either an intervention or waitlist control condition. Girls and coaches in the intervention condition will complete three 60-minute sessions over three consecutive weeks. The primary outcome will be the immediate change in girls’ body esteem, with secondary outcomes assessing the immediate and short-term changes in girls’ body appreciation, self-objectification, attuned self-care, sports enjoyment, and affect.

**Discussion:**

This research is the first to utilise an international multi-stakeholder partnership to co-create and evaluate an intervention that addresses the intersection of girls’ body image and sport experiences. The theoretical and methodological considerations of this research have led to a feasible intervention and trial protocol, and if proven effective, BCA may assist in reducing the global gender disparity in sports participation.

**Trial registration number:**

NCT05594524, registered 25^th^ October 2022.

**Supplementary Information:**

The online version contains supplementary material available at 10.1186/s12889-023-16360-w.

## Background

Globally, girls disengage and drop out of sport at a significantly earlier age and higher rate than boys [1]. Numerous biopsychosocial factors have been found to spur these rates, many of which intersect with body image [2]. Body image is broadly defined as the way an individual thinks, feels and behaves towards their body, including its appearance and how it functions. Girls’ body image in sports is influenced by biological (e.g., puberty), psychological (e.g., comparison making), and social (e.g., gender stereotypes, appearance and athletic ideals, appearance-based teasing) factors that are both universal (e.g., puberty, comparison making, appearance-teasing) and context-specific (e.g., gender stereotypes, athletic ideals) [2]. During puberty, girls experience rapid physical changes (i.e., larger breasts, wider hips, weight gain) that are both commodified and criticised by the sports community, and by society more broadly [3, 4]. Specifically, female bodies are over-evaluated and objectified through revealing sports attire, appearance-oriented media coverage and harassment from male peers; simultaneously, girls’ maturing bodies can be perceived as problematic and disadvantageous to sports (e.g., the perception that developed and larger bodies are associated with poorer performance [5, 6]). As a result, many girls are encouraged by socio-cultural agents (e.g., peers, coaches, and the media) to under fuel and over train their bodies to suppress the natural development of the female body [7]. Subsequently, a growing number of girls disengage from sports due, in part, to this undue pressure, and those who remain are at increased risk of developing body dissatisfaction, disordered eating, and/or an eating disorder, particularly those participating in appearance- (e.g., gymnastics, figure skating) or weight-sensitive sports (e.g., cross-country, rowing) [6, 8–11].

### Targeting the intersection of girls’ body image and movement experiences

To date, numerous interventions have sought to address the intersection between girls’ body image and movement experiences (i.e., physical activity, exercise, and sport), as a means for reducing gender disparities in movement participation; however, in a recent systematic review and meta-analysis these approaches were marginally effective, if not ineffective. This is, in part, due to theoretical and methodological limitations within and across the 12 studies pertaining to girls. For instance, a majority of interventions utilise a risk reduction framework, whereby the primary aim is to reduce girls’ body dissatisfaction through increased movement (e.g., aerobic dance class). In only focusing on, and alleviating, body dissatisfaction, girls, at best, develop a neutral relationship with their bodies (e.g., *“I neither like or dislike my body while dancing”*), rather than a deeper acceptance, appreciation, and respect for what their body can do and experience while participating in sport (e.g., *“I’m grateful for my legs. They propel me in the air when I’m dancing”*; *“I’m thankful that my body can heal itself from injury”*) [12]. This latter perspective reflects positive body image and embodiment theories [13–15], which offer a more nuanced and holistic view of one’s relationship with their body, beyond simply being satisfied with how it looks.

To our knowledge, only one intervention has explicitly utilised positive body image and embodiment principles to address the intersection of girls’ body image and movement experiences (Healthy Body Image [HBI]; [16]). Specifically, HBI comprises three 90-min workshops, which address negative and positive body image (workshop one), media literacy (workshop two), and healthy lifestyles (workshop three). Evaluations of HBI in Norwegian high schools (*N* = 2,446 girls (57%) and boys; *M*_*age*_ = 16 years) [17, 18], found improvements in girls’ positive body image [17], but not their movement levels [18]. Collectively, these findings indicate that HBI is an effective positive body image intervention for adolescent girls; however, it may be ineffective at addressing the intersection between girls’ body image and movement. This is likely due to a disconnect between intervention content and movement contexts. That is, the intervention techniques address general positive body image concepts (e.g., How can changing from an aesthetic to a functional focus improve body experiences and healthy lifestyles?), rather than relating and applying these concepts to movement contexts and experiences (e.g., What is my body able to do and experience when playing sport, and why is this important?). To our knowledge, an intervention of this nature has yet to be developed.

### Co-creation and task shifting intervention delivery

Another key limitation of existing interventions is the lack of community-based participatory research (CBPR) principles and practices incorporated into the development and/or selection of an intervention [19]. For example, to our knowledge, a majority of existing studies in this space did not consult with key stakeholders who would be involved in, or impacted by, the intervention during community dissemination and implementation (e.g., girls, coaches, sport organisations, and community or industry partners). This oversight has implications for intervention feasibility, including its acceptability, implementation, integration, and efficacy [20]. The use of CBPR is recognised as a key element of effective intervention development and is mandatory in some peer-reviewed communities (e.g., journal submission requirements). Relatedly, a majority of the abovementioned studies used exercise, body image, and/or mental health specialists to deliver the intervention to girls. Reliance on specialists for dissemination and implementation also has implications for intervention feasibility and scalability [21]. Task shifting to non-specialists has proven effective, but without some of the financial and human resource costs accompanied with the use of specialists [21].

Additional advantages of task shifting intervention delivery to non-specialists and, more specifically, to influential members within sport communities, for instances coaches, include: 1) coaches have an existing relationship with girls, and are likely to have good group management skills and a passion for supporting young people; 2) coaches become skilled with the knowledge and strategies to effectively and sustainably deliver a body image intervention within sport contexts; 3) coaches are influential members within their communities, and once trained, could use their newly acquired knowledge and strategies to influence other system-levels within these communities (e.g., policy change for girls’ sport); and 4) coaching is a transient profession, which requires travel to, and interacting with, other sport communities (e.g., tournaments, professional development), which might allow for further dissemination of core learnings and messages. Therefore, developing a feasible intervention that coaches like and find easy to disseminate and implement within their communities will increase their likelihood of sharing this resource with other coaches and athletes who would benefit.

### The present paper

This paper outlines the co-creation, initial piloting and protocol for a cluster randomised controlled trial of Body Confident Athletes (BCA); an in-person coach-led intervention that aims to increase girls’ positive body image and sports enjoyment. The co-creation of this intervention was guided by the framework for developing complex interventions (see Table 1; 23,24), with the procedures for the pilot and the cluster randomised controlled trial informed by the Standard Protocol Items: Recommendations for Interventional Trials (SPIRIT; see Table 2), the Consolidated Standards of Reporting Trials (CONSORT; [22]), and the extension to cluster randomised trials (CONSORT; [23]). The SPIRIT and CONSORT checklists are provided in the Supplementary materials.

**Table 1:** Guide for developing complex interventions – key steps and considerations

Action	Recommended Steps & Considerations	Steps Conducted for Body Confident Athletes
**1. Plan the development process**	1. Identify the problem to be targeted and refine understanding of it throughout the process (iterative as literature emerges). 2. Assess whether the problem is a priority. 3. Consider which aspects of the problem are amenable to change. 4. Ask whether a new intervention is really needed and if the potential benefit of the new intervention. justifies the cost of development 5. Determine the time needed to undertake intervention development. 6. Obtain sufficient resources/funding for the intervention development study. 7. Draw on one or more of the many published intervention development approaches, recognising that there is no evidence about which approach is best and apply flexibly depending on the problem and context. 8. Involve stakeholders during the planning process (see next Action). 9. Produce a protocol detailing the processes to be undertaken to develop the intervention.	1. The problem was defined as: Girls disengage and drop out of sports due to body image concerns at a disproportionate rate to boys. 2. Assessed the priority of the problem via: a). Scientific literature b). News and social media; c). Government policy. 3. Reviewed existing and monitored new literature into girls’ body image experiences in sport contexts. 4. Body image is a malleable trait that can be improved through concerted intervention. Therefore, it was proposed that a body image intervention that is specific to sport contexts would enhance girls’ body image and sports enjoyment; thus, reducing the gender disparity in sports participation. 5. Our systematic review and meta-analysis found that existing interventions that address the intersection of girls’ body image and movement experiences, including sport, were marginally effective at improving body image and ineffective at improving movement-related variables. Key limitations included: a). A majority of interventions used a risk reduction framework (i.e., reduce body dissatisfaction), rather than addressing both risk and protective factors (i.e., reduce risk, while enhancing one’s relationship with their bodies beyond satisfaction). b). No interventions incorporated CBPR into the development and/or selection of an intervention; thus, implicating intervention feasibility, including acceptability, implementation, integration, and efficacy. 6. A detailed research proposal, including project aims, deliverables, protocols (e.g., where and how to involve stakeholders), and timelines were presented to industry partners for review and approval. 7. The proposal was approved and subsequently funding was obtained via two industry partners.
**2. Involve stakeholders including those who will deliver, use and benefits from the intervention**	1. Work closely with relevant stakeholders throughout the development process: patients, the public, the target population, service providers, those who pay for health and social services or interventions, policymakers, and intervention design specialists. 2. Develop a plan at the start of the process to integrate public and patient involvement into the intervention development process. 3. Identify the best ways of working with each type of stakeholder, from consultation through to coproduction, acknowledging that different ways may be relevant for different stakeholders at different times. 4. Use creative activities within team meetings to work with stakeholders to understand the problem and generate ideas for the intervention.	1. A global multi-stakeholder partnership was formed between academics, community, and industry partners. The partnership provided access to the target audience and community (i.e., girls, coaches, sport organisations). 2. Stakeholder input was strategically approached during the research planning phase; thus, ensuring that appropriate stakeholders were approached and that their time and expertise were used efficiently. 3. Creative and efficient ways of working were identified for the respective stakeholders (e.g., weekly meetings between core partners; focus groups with girls and coaches).
**3. Bring together a team and establish decision making processes**	1. Include within the development team individuals with relevant expertise: in the problem to be addressed by the intervention including those with personal experience of the problem, in behaviour change when the intervention aims to change behaviour, in maximising engagement of stakeholders and with a strong track record in designing complex interventions. 2. It may be hard to make final decisions about the content, format, and delivery of the intervention, so only some team members may do this. There is no consensus about the size or constituency of the team that makes these final decisions, but it is important early on to agree a process for making decisions within the team.	1. A project team was acquired that had relevant expertise to ensure intervention feasibility, including acceptability, implementation, integration, and effectiveness. 2. Review periods were incorporated throughout the research timeline, which allowed for key stakeholders to review the intervention content, format, and structure.
**4. Review published research evidence**	1. Review published research evidence before starting to develop the intervention and throughout the development process for example, to identify existing interventions, to understand the evidence base for each proposed substantive intervention component. 2. Look for, and consider, evidence that the proposed intervention may not work in the way intended.	1. Prior to intervention development, a systematic review and meta-analysis was conducted to identify existing interventions that address the intersection of body image and sport among girls, and key intervention, participant, and methodological features that impact intervention effectiveness. Findings from this research further refined the proposed intervention.
**5. Draw on existing theories**	1. Identify an existing theory or framework of theories to inform the intervention at the start of the process, for example, behaviour change or implementation theory. 2. Where relevant, draw on more than one existing theory or framework of theories for example, both psychological and organisational theories.	1. Theories pertaining to positive and negative body image and embodiment informed the theoretical framework of the new intervention 2. Following the systematic review and meta-analysis and focus groups with girls, an intervention framework was developed which outlined the intervention themes and underpinning theories, and the format and structure. The theoretical perspectives and frameworks underpinning the intervention activities, included: a). The Tripartite Influence Model for Body Image [24]; b). Positive Body Image and Embodiment Theories [13–15, 25, 26], including Agency and Functionality, Body Connection and Comfort, Body Appreciation, Body Functionality, Resisting Objectification, and Attuned Exercise; c). Cognitive Dissonance [27].
**6. Articulate programme theory Note. A programme theory describes how a specific intervention is expected to lead to its effects and under what conditions**	1. Develop a programme theory. The programme theory may draw on existing theories. Aspects of the programme theory can be represented by a logic model or set of models. 2. Test and refine the programme theory throughout the development process.	1. Programme Theory: If given the opportunity to play sports, girls face unique body image-related challenges that impede their participation and enjoyment, relative to boys. Their sport experience is hindered, if not halted, by the promotion of harmful gender stereotypes, unrealistic appearance and athletic ideals, the sexual objectification of female athletes’ bodies, and the vilification of girls’ physical competence. This relentless evaluation of female bodies can lead to appearance preoccupation, surveillance and dissatisfaction among girls, which in turn can diminish their concentration, performance and enjoyment of sports. Lastly, these negative sport experiences are further compounded by coaches and sports communities being ill-equipped to deal with these matters. Body Confident Athletes will educate girls about and how to challenge gender stereotypes, and unrealistic appearance and athletic ideals within sport contexts (e.g., body talk). It will also upskill them with the knowledge and strategies to develop a deeper acceptance, appreciation, and respect for their body, and what it can experience when playing sports, beyond what it looks like. That is, girls’ attention will move away from “What does my body look like during sport?” and towards “What can my body do and experience”, which in turn will increase girls’ body connection and comfort. 2. The programme theory has been refined throughout the development process, including the consideration and incorporation of new literature, and review sessions with girls, coaches, community, and industry partners.
**7. Undertake primary data collection**	1. Use a wide range of research methods throughout, for example, qualitative research to understand the context in which the intervention will operate, quantitative methods to measure change in intermediate outcomes.	1. A wide range of research methods were utilised and will continue to be utilised throughout this research, including: a. Systematic reviews and meta-analyses; b. Qualitative assessment tools (e.g., semi-structured focus groups); c. Quantitative assessment tools (e.g., validated measures to assess change in primary and secondary outcomes in the pilot and main trials); d. Randomised controlled trials, including an initial pilot (e.g., assessing initial feasibility) followed by a main trial (e.g., assessing efficacy).
**8. Understand the context**	1. Understand the context in which the intervention will be implemented. Context may include population and individuals; physical location or geographical setting; social, economic, cultural, and political influences and factors affecting implementation, for example, organisation, funding, and policy.	1. Both formal and informal information gathering steps were undertaken to better understand the context under which the intervention would be implemented and integrated into sport contexts. This included: a. Project scoping and planning meetings with topic experts from the core partnership group (i.e., academics, community, and industry partners); b. Systematic review and meta-analysis of existing interventions, and identifying optimum intervention features (e.g., number and length of sessions); c. Semi-structured focus groups with girls and coaches from six countries, and qualitative surveys with young women from an additional seven countries. Participants provided insights into country and cultural nuances needing consideration in the new intervention, including gender norms (e.g., girls are not expected to play sport), socioeconomics (i.e., access to resources; facility standards), religiosity (e.g., uniform accommodation). Where appropriate, this information has been considered and incorporated into the intervention. Alternatively, these considerations will be signposted and explained in a localisation toolkit, which will enable those implementing the intervention to adapt the content/delivery accordingly, while retaining the evidence-based mechanisms that make the intervention effective.
**9. Pay attention to future implementation of the intervention in the real world**	1. From the start, understand facilitators and barriers to reaching the relevant population, future use of the intervention, ‘scale up’, and sustainability in real world contexts.	1. From the outset, the research designs for both the pilot and main randomised controlled trials have been considered and refined, as these will reflect real-world dissemination, implementation, and integration. For example, the delivery of Body Confident Athletes is task shifted to coaches, as they were identified as both influential community members (e.g., have close relationships with girls) and can permeate their knowledge and skills to multiple teams and system levels. To ensure sustainability was maintained as a key objective, the RE-AIM model for evaluating interventions was employed, such that the intervention was assessed on a number of metrics beyond the standard ‘efficacy’ (i.e., acceptability, integration etc.).
**10. Design and refine the intervention**	1. Generate ideas about content, format, and delivery with stakeholders 2. Once an early version or prototype of the intervention is available, refine or optimise it using a series of iterations. Each iteration includes an assessment of how acceptable, feasible, and engaging the intervention is, including potential harms and unintended consequences, resulting in refinements to the intervention. Repeat the process until uncertainties are resolved 3. Check that the proposed mechanisms of action are supported by early testing.	1. Intervention development was an iterative process conducted between academic experts in body image and sport and key stakeholders, including girls, coaches, community, and industry partners: a. Initially, an intervention framework was developed, which outlined the intervention theory and aims, key intervention features (e.g., number and length of sessions; activities), and the primary and secondary outcomes; b. Next, the framework was reviewed and signed off by the academic, community, and industry partners; c. Next, the framework was expanded upon, with an initial intervention prototype developed (e.g., a fully formed session guide for coaches; an athletes’ workbook); d. Next, the intervention prototype was reviewed and signed off by the academic, community, and industry partners, and then by a small group of girls and coaches; e. Next, minor modifications were made to the intervention (e.g., changing terminology for greater comprehension); f. Next, the intervention was pilot tested in a small-scale cluster randomised controlled (pilot) trial, where further data was gained on intervention feasibility, including girls and coaches’ acceptability, intervention implementation, and integration; g. To finish, the intervention will be tested in large-scale cluster randomised controlled trial, where similar data will be captured to confirm acceptability, implementation, and integration, as well as efficacy
**11. End the development phases**	1. There are no established criteria for stopping the intensive development phase and moving on to the feasibility/pilot or evaluation phases. The concepts of data saturation and information power may be useful when assessment of later iterations of the intervention produces few changes. 2. Describe the intervention to facilitate transferability of an intervention outside the original team and location in which it was developed. 3. Write up the intervention development process so that judgements can be made about the quality of the process, links can be made in the future between intervention development processes, and the subsequent success of interventions, and others can learn how it can be done.	1. The large-scale cluster randomised controlled trial will provide additional data for the intervention feasibility, including acceptability, implementation, and integration. This data will be used to further refine the intervention. 2. A comprehensive overview of the intervention development process is provided in this study protocol, which will be available via an open-access journal. The intervention materials will also be freely available to the public, and therefore will be available for review and potentially additional evaluations beyond the current research team.

**Table 2:** Schedule of enrolment, interventions, and assessments according to the Standard Protocol Items: Recommendations for Interventional Trials

		**STUDY PERIOD**				
	**Enrolment**	**Post-allocation**				
**TIMEPOINT:**	***-t***_1_	***t***_1_	***Intervention***	***t***_***2***_	***t***_***3***_	***t***_***4***_
**TIME:** *Days from baseline assessment*	***-21***	***0***	** + *1 –* + *21***	** + *22***	** + *50***	** + *106***
**ENROLMENT:**						
*Eligibility screen*	X					
*Parental consent*	X					
*Participant informed assent*	X					
*Randomisation/ allocation*	X					
**INTERVENTIONS:**						
*Body Confident Athletes*						
*Waitlist Control*						
**ASSESSMENTS *Baseline variables*:**						
*Age*	X	X		X	X	X
*Gender*	X	X		X	X	X
*Ethnicity*		X		X	X	X
*Race*		X		X	X	X
*State and region of residence*		X		X	X	X
***Primary Outcome measures:***						
*Trait body image (BESAA)*		X		X	X	X
***Secondary outcome measures***						
*Body Appreciation*		X		X	X	X
*Attuned Self-Care*		X		X	X	X
*Resisting Objectification*		X		X	X	X
*Sports Enjoyment*		X		X	X	X
*Positive and Negative Affect*		X		X	X	X
*Intervention Fidelity (Coaches’ Delivery and Athletes’ Comprehension)*				X		
*Intervention Attendance*				X		

**Fig. 1:**
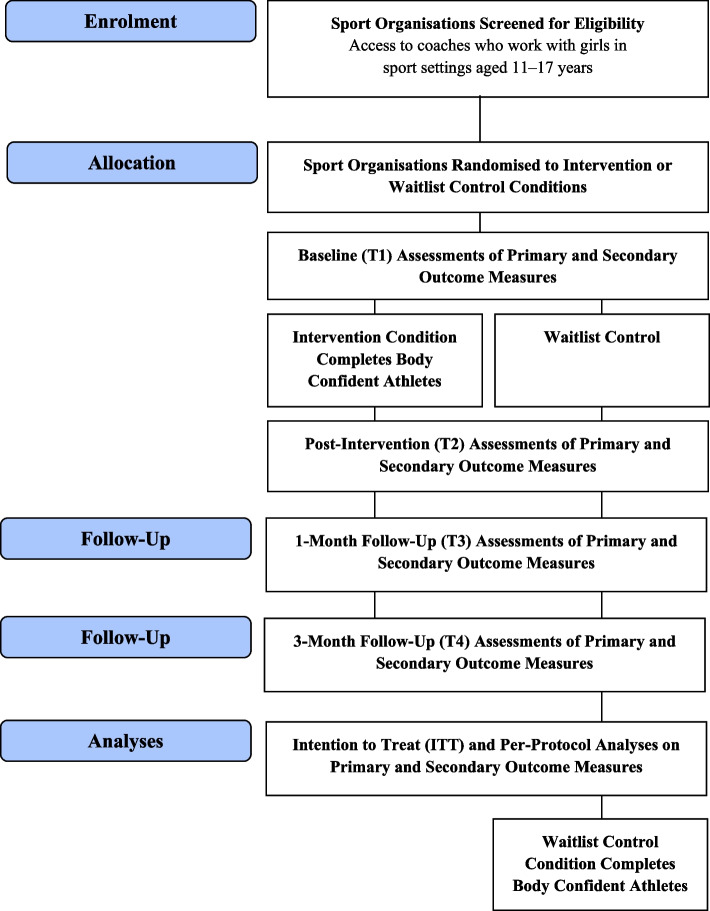
The main trial research design according to the Consolidated Standards of Reporting Trials (CONSORT)

Findings from the co-creation (see section Cluster randomised controlled (Pilot) trial) and initial pilot (see section Co-creating the intervention) informed the final protocol for the cluster randomised controlled trial (Fig. 1), which will compare BCA to a waitlist control condition (i.e., participate in sport as usual). This is a pragmatic trial conducted in settings similar to the “real world” (i.e., adopts a sport setting, coach-led delivery, delivered during typical sport practice, no strict inclusion or exclusion criteria for athletes, comparison to usual sports participation) [28]. Participants in the intervention condition will complete three 60-minute sessions over three consecutive weeks. At completion of the final follow-up survey, girls and coaches in the control condition will participate in the intervention; however, their participation will not be monitored or evaluated.

Assessment of primary (body esteem) and secondary (body appreciation, self-objectification, attuned self-care, sports enjoyment, and affect) outcomes will be conducted at baseline (T1), post-intervention (T2), 1-month follow-up (T3), and 3-month follow-up (T4). Fidelity assessments will be conducted on both coaches’ delivery of the intervention, as well as girls’ comprehension of the intervention content. It is hypothesised that girls randomised into the intervention condition will experience greater improvements in body image (body esteem, body appreciation, attuned self-care, resisting objectification), sports enjoyment, and positive and negative affect at post-intervention, 1-month, and 3-month follow-up, relative to the waitlist control condition.

## Method

### Community-Based Participatory Research (CBPR)

This research adopted the CBPR principles as outlined by Collins and colleagues [19], who describe this framework as an inclusive and flexible approach to equitably involving key stakeholders in research. First and foremost, an international multi-stakeholder partnership was formed and maintained between girls, coaches, academics, and community and industry partners throughout the entirety of this research. Second, the target audience (i.e., girls), as well as those impacted by the new intervention (i.e., coaches) were consulted on throughout this research. For instance, initially, we consulted with girls (*n* = 91) on their experience of the said issue (e.g., body image concerns in sport), along with their preferences for how to address these concerns in a new intervention. Details and findings from this research phase are reported elsewhere [29]. From here, an initial intervention prototype was developed, whereby a smaller group of girls (*n* = 10) and coaches (*n* = 4) were invited to provide feedback on the intervention content, structure, and format. Next, a separate group of girls (*n* = 26) and coaches (*n* = 7) participated in a pilot study in the US (see section Cluster randomised controlled (Pilot) trial), which assessed the intervention feasibility, including acceptability, implementation, integration, and limited efficacy testing [20], as well as the feasibility of conducting a two-armed, cluster randomised controlled trial. Girls and coaches participating in these two phases were recruited via a sport organisation, Laureus Sport for Good.

### Co-creating the intervention

A *combination approach* to intervention development was utilised [30, 31], whereby the existing evidence base on girls’ body image, sports, and mental health and well-being were coupled with CBPR to inform intervention content, structure, format, dissemination, and implementation. The key steps associated with this approach are reported in Table 1. To increase intervention scalability, this research adopted a cross-national lens from the outset and intersected with all elements of the research, including an initial systematic review and meta-analysis (e.g., considering existing interventions from all countries), stakeholder input (e.g., consultation with girls from 13 countries [29]), and intervention materials (e.g., development of a localisation toolkit for intervention adaptation and localisation for different countries).

The intervention content (e.g., session topics and activities) targets both risk and protective factors for body image. Topics and activities were taken from existing evidence-based body image interventions and then adapted for, and applied to, sport-specific settings and scenarios. Meanwhile, the intervention structure (i.e., four key sections within each session) and format (i.e., five 60-min sessions) were informed by previous reviews into the optimum intervention features for addressing body image and/or movement among adolescent girls [32–35]. A summary of the intervention sessions, and the associated theories, learning objectives, and learning experiences are reported in Table 3.

**Table 3:** Body confident athletes intervention sessions, underpinning theories, learning objectives, and learning experiences

Sessions	Underpinning Theories	Session Rationale	Learning Objectives	Learning Experiences
Body Talk in Sport	1. **Body Talk **[36]: Body talk is a form of dialogue that reinforces harmful gender stereotypes, appearance and athletic ideals within sport, and society more broadly. 2. **Tripartite Influence Model of Body Image **[24]: Body talk between friends, teams, families, and in the media (e.g., social media comments) reinforces harmful gender stereotypes and appearance and athletic ideals. 3. **Cognitive Dissonance **[27]: There are individual and societal costs and consequences to engaging in body talk. Cognitive dissonance strategies help to challenge body talk and reduce these costs.	This session sets the scene for athletes by introducing them to body talk, and how these conversations can reinforce harmful gender stereotypes and appearance and athletic ideals, and negatively impact girls’ sport experiences.	1. Understand what is meant by body talk, who engages in it and where. 2. Recognise the negative impact that body talk can have on athletes’ body image and sport experiences. 3. Develop strategies for challenging body talk and creating a Body Talk Free Zone.	1. **The Game Plan**: Introduce the programme and outline session one. 2. **The Knowledge**: Through group discussion, introduce the concept of body talk, where it occurs and its impact on girls’ body image and sport experiences. 3. **The Skills**: In small groups, brainstorm counteractive responses (“comeback challenges”) for body talk statements. 4. **The Final Score**: Finish the session with a recap of learnings, a weekly homework task (i.e., identify and challenge body talk), and a body connection activity, the “Three-Step Sense Check”.
What Our Bodies Experience in Sport	1. **Agency and Functionality **[15]**; Body Functionality **[25]**; Body Appreciation **[37]: Recognise, accept, and appreciate what the body can experience, and perhaps do differently to others in sport, including: physical capacities (e.g., outrun an opponent), internal processes (e.g., heal from an injury), bodily senses and perceptions (e.g., reflexes), creative endeavours (e.g., self-expression through dance), communication (e.g., celebrating a team member’s goal), and self-care (e.g., mood regulation).	In session two, athletes learn how to focus on and accept what their bodies can do and experience during sport, rather than on what it looks like. By shifting their focus, athletes are more likely to accept and appreciate their different abilities and be in tune with what their body needs when playing sport.	1. Recognise the difference between focusing on what their body can experience vs. what their body looks like when playing sport. 2. Understand how this way of thinking improves their body image and sport experiences. 3. Develop strategies to improve their awareness and gratitude for what their body can do and experience.	1. **The Game Plan**: Recap session one, homework check-in (e.g., did athletes challenge body talk?), and introduce session two. 2. **The Knowledge**: Through group discussion, introduce the concept of body functionality (e.g., recognising, accepting, and appreciating what the body can do and experience, beyond what it looks like) 3. **The Skills**: In an individual writing task, focus on what the body can do and experience when playing sports and why this is important to the athlete. Sometimes this might mean focusing on what our bodies do differently to others. 4. **The Final Score**: Finish the session with a recap of learnings, a weekly homework task (i.e., practise the writing task), and a body connection activity, the “Three-Step Sense Check”.
Listening to Our Bodies in Sport	1. **Attuned Exercise **[26]: Moving the body in ways that promote mindfulness, body connection, body responsiveness, self-compassion, self-acceptance, and joy. In feeling physically and psychologically safe (i.e., the movement does not harm the body), individuals can focus on becoming more aware, connected, and responsive to their body and come to experience joy through sport. 2. **Body Connection and Comfort **[15]: Reflect on the quality of the connection with the body and the degree of comfort in the body when playing sport. This includes engaging in constructive self-talk.	To finish the programme, athletes learn the importance of listening to their bodies, as well as the potential consequences of not listening to them. Athletes engage in a practical task, where they practise listening to their body’s sensations and if necessary, responding to these sensations to meet the body’s needs	1. Identify the type of sensations our bodies experience and why. 2. Understand the importance of listening and attending to our bodies’ needs. 3. Develop strategies that help us to connect with our bodies and if necessary, respond to these sensations to meet the body’s needs.	1. **The Game Plan**: Recap session two, homework check-in (e.g., did athletes practise the writing tasks?), and introduce session three. 2. **The Knowledge**: Through group discussion, athletes explore different bodily sensations, and the benefits of, and/or consequences of not, listening to these sensations while playing sport. 3. **The Skills**: In an individual movement task (e.g., shooting on goal; practising a ball trick), athletes practise listening to and attending to their bodies’ sensations. 4. **The Final Score**: Finish the programme with a recap of learnings and a body connection activity, the “Three-Step Sense Check”.
Gender Stereotypes in Sport (booster session one)	1. **Tripartite Influence Model of Body Image **[24]: Understand and identify how society (family, friends, the media) create and perpetuate harmful stereotypes about girls and women in sport. 2. **Cognitive Dissonance **[27]: Understand and identify the costs that these stereotypes have on girls and women, and their participation in sport. 3. **Body Appreciation **[37]** and Resisting Objectification **[15]**:** By creating more realistic and inclusive ideas of beauty and athleticism, as well as appreciating our bodies for what they can do, we protect ourselves by rejecting unrealistic appearance ideals.	If teams have capacity for delivering 3 + sessions, Gender Stereotypes in Sport can be delivered as the first session of the programme (i.e., before Body Talk in Sport). Athletes explore how female athletes’ bodies are represented in society (i.e., through language and images), and how these representations reinforce harmful gender stereotypes, appearance, and athletic ideals.	1. Understand society’s role in reinforcing potentially harmful gender stereotypes for girls, women, and gender diverse people in sport 2. Identify the costs that these stereotypes have on people and their engagement in sport. 3. Develop strategies for challenging these stereotypes.	1. **The Game Plan**: Introduce the programme and outline session one. 2. **The Knowledge**: Through group discussion, introduce gender stereotypes in sport and how these can be harmful to girls’ body image and sport experiences. 3. **The Skills**: Brainstorm ways to challenge stereotypes during a paired movement activity (e.g., Every time you execute a simple skill from your sport [catch and throw in lacrosse], you need to come up with a new way to challenge gender stereotypes). 4. **The Final Score**: Finish the session with a recap of learnings, a weekly homework task (i.e., challenge gender stereotypes in sport), and a body connection activity, the “Three-Step Sense Check”.
Advocacy in Sport (booster session two)	1. **Cognitive Dissonance **[27]**:** Taking individual and group action that challenge harmful societal attitudes and behaviours that perpetuate inequalities for girls and women in sport, in particular gender stereotypes, and appearance and athletic ideals. 2. **Socioecological Model for Addressing Girls’ Body Image in Sport **[2]: To create equitable sport environments that allow girls and women to reach their full potential, social changes need to occur across all levels of society, including individual, interpersonal, organisational/ environmental, and societal.	If teams have capacity for delivering 3 + sessions, Advocacy in Sport can be delivered as the last session of the programme (i.e., after Listening to Our Bodies in Sport). Athletes learn to use their new knowledge and skills to advocate for change in girls' and women's sport. Change will centre around body image-related issues; however, advocacy skills learned in this session can be extended to other sport experiences or girls' and women's rights more broadly	1. Identify the body image-related challenges and inequalities that girls and women may face in sport. 2. Understand how these challenges and inequalities can negatively impact the future of girls’ and women’s sport. 3. Develop advocacy strategies for addressing these inequalities as an individual and community.	1. **The Game Plan**: Recap the previous session, homework check-in (e.g., did athletes practise the movement activity?), and introduce the session. 2. **The Knowledge**: Through group discussion, discuss the body image-related challenges and inequalities that girls and women may face in sport and how these impact the future of girls’ and women’s sport. 3. **The Skills**: In small groups, brainstorm ways to overcome these inequalities across different levels of the sport ecosystem (e.g., individuals, communities, organisations, government). 4. **The Final Score**: Finish the programme with a recap of learnings and a body connection activity, the “Three-Step Sense Check”.

Where theories relate to general contexts and settings (e.g., Body Talk [27]; Body Appreciation [36]), the theory has been related and applied to a sports context

**Fig. 2:**
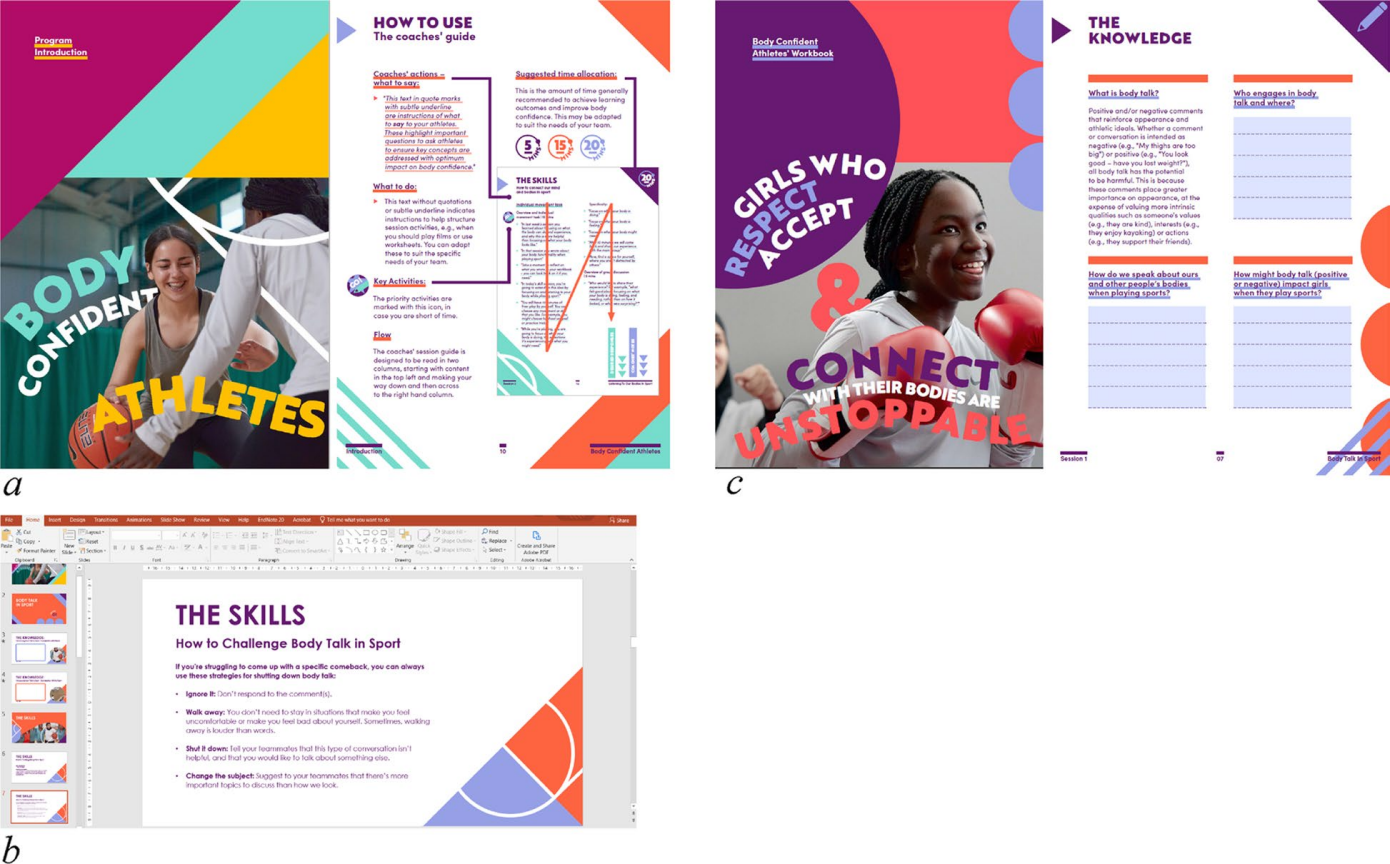
Screenshots of the Body Confident Athletes materials including the coaches’ session guide (**a**), presentation deck (**b**), and athletes’ workbook (**c**)

The intervention materials comprised of: 1) a coaches’ session guide that provides step-by-step instructions on how to deliver each session; 2) 3 × 5-min coaches’ training videos that contextualise body image in sport and strategies for how to deliver the programme effectively; 3) a presentation deck containing visual stimuli that aims to facilitate discussions during the sessions; and 4) an athletes’ workbook where girls can privately respond to questions and discussions addressed in the sessions. A screenshot of the materials is provided in Fig. 2.

Lastly, a localisation toolkit will sit alongside the intervention materials, and will support dissemination partners on how to adapt and localise content such that it is culturally relevant. For instance, using examples of local and national sports women; acknowledging cultural norms that impact girls’ body image and sports participation (e.g., parents not allowing girls to wear sports attire in India); being aware of and sensitive to religious beliefs and practices and how this impacts girls sport experiences. This content was informed by the initial consultation with girls and the cross-national considerations they raised [29].

### Cluster randomised controlled (Pilot) trial

#### Pilot procedure

The pilot was, and the main trial will be, conducted in the US. This was a collaborative decision made by the multi-stakeholder partnership group; a decision that was informed by existing connections and resources of the partnership, which would increase the feasibility of conducting the trial within the intended timeline. The aim of the pilot was to test the preliminary feasibility of the intervention (acceptability, implementation, integration, and limited efficacy) [20], as well as the feasibility of conducting a two-armed, cluster randomised controlled trial in the US. The pilot trial was held between May–October 2022 (May–July = recruitment; August–October = data collection), with findings informing the final version of the intervention that would be trialled (i.e., content, structure, and format), as well as the protocol for the main trial.

Initially, organisations were randomised into either the intervention or waitlist control condition. Following this, girls and coaches in both conditions completed a baseline assessment containing self-report measures of demographics (i.e., age, ethnicity, coaching experience or playing experience). Girls also completed self-report measures of trait body image (primary outcome; [38]) and sports enjoyment (secondary outcome; [39]), which informed preliminary efficacy. Next, those in the intervention condition completed five 60-minute sessions over five consecutive weeks. At post-intervention, girls in both conditions but only coaches in the intervention condition completed a second survey. All girls completed post-intervention assessments of trait body image and sports enjoyment, and girls and coaches in the intervention condition completed assessments on intervention acceptability (13 items; e.g., *The programme covered topics that are important to my athletes* [coaches]; *I would recommend the Body Confident Athletes programme to other girls* [girls]), with coaches also providing assessments on intervention structure and integration (7 items; e.g., *Our club or school could sustainably continue offering this programme to girls [e.g., to newcomers; in our outreach programmes]*).

Intervention fidelity was also assessed via session recordings. Specifically, coaches were scored on the accuracy with which they delivered the intervention sessions, and girls’ responses were assessed for relatedness to the key learning objectives in *The Knowledge* and *The Skills* sections of the sessions. At completion of the post-intervention assessments, girls and coaches in the control condition were provided with the intervention materials; however, their participation was not evaluated. To compensate participants for their time, girls and coaches received an electronic voucher to the value of US$60.

#### Pilot findings

Initially, eight organisations were recruited for participation in the pilot trial, with four organisations dropping out at baseline (i.e., due to staff turnover or scheduling clashes). Girls and coaches from four organisations saw the pilot to completion, including completing baseline and post-intervention assessments, and completing five 60-minute sessions over five consecutive weeks (see supplementary Fig. 1 for design and participant flow). A total of 26 girls participated in the pilot (*M*_*age*_ = 12.73 [*SD* = 1.99];* n* = 13 in intervention; *n* = 13 in control). Nineteen girls completed baseline assessments (*n* = 10 in intervention; *n* = 9 in control), with 12 providing post-intervention data (*n* = 7 in intervention; *n* = 5 in control). A majority of girls identified as White (34.61%, *n* = 9), followed by 23.07% (*n* = 6) as Hispanic, 19.23% (*n* = 5) as Black, 11.54% (*n* = 3) as Asian, 7.69% (*n* = 2) as multi-racial, and one preferred not to say. Participants had been a member of their sport organisation for an average of 3.38 years (*SD* = 1.52). Girls in the two arms did not significantly differ on any of the demographic variables. A total of seven female coaches (*M*_*age*_ = 29.71 [0.92]; *n* = 4 in intervention; *n* = 3 in control) participated in the pilot. All coaches completed baseline assessments; however, only three coaches from the intervention condition provided post-intervention data. Three coaches were Black (42.85%), three were White (42.85%), and one was multi-racial (14.28%). Coaches had been a member of their sport organisation for an average of 3.00 years (*SD* = 1.40). Coaches in the two arms did not significantly differ on any of the demographic variables.

With respect to intervention fidelity (i.e., was the intervention delivered as intended), a random subset of sessions was autonomously assessed by two raters to check for inter-rater reliability. The remaining sessions were equally split and assessed by only one rater. We calculated Cohen’s κ as a measure of agreement for all the categorical items (Yes/No items for coaches’ fidelity assessment on each section of the sessions; *The Game Plan*, *The Knowledge*, *The Skills*, *The Final Score*) and ICC for all the ordinal items (0–100 items for athlete’s fidelity assessment on *The Knowledge* and *The Skills*). Overall, Cohen’s κ was 0.79 (*z* = 7.15, *p*-value < 0.001), indicating substantial agreement between raters. Similarly, the ICC was 0.88 (*F*(7,8) = 15.30, *p* < 0.001), suggesting excellent between-raters reliability. Overall, coaches scored 75.35% on fidelity of intervention delivery. Specifically, coaches scored 95.83% on *The Game Plan* section, 67.06% on *The Knowledge* section, 70.83% on *The Skills* section, and 67.70% on *The Final Score* section. These scores are comparable to other multi-session body image interventions delivered by non-specialists [40, 41]. Girls also demonstrated a high level of intervention comprehension, with an overall score of 88.68%. Specifically, girls scored 91.66% on *The Knowledge* section and 85.71% on *The Skills* section. With respect to intervention adherence, of the 13 girls initially randomised to the intervention, seven girls attended Session 1 and 2 (53.84%), four attended Session 3 (30.77%), three attended Session 4 (23.07%), and six attended Session 5 (43.15%). Overall, two athletes (15.38%) attended all five sessions.

With respect to intervention acceptability and integration, coaches scored both the intervention materials and topics (*M* = 4.21 out of 5 [*SD* = 0.87]) and the potential integration of the intervention highly (*M* = 4.00 out of 5 [*SD* = 0.62]). Similarly, athletes reacted positively to the intervention (*M* = 4.18 out of 5 [*SD* = 0.38]). Qualitative feedback from girls and coaches was also obtained. Due to the small sample of responses, formal qualitative analyses were not conducted. Overall, girls and coaches felt the intervention created a space for girls to discuss important and relevant topics. Key areas for improvement included greater accessibility and representation of girls with Physical Disabilities who play sport, and a printer friendly version of the coaches’ session guide.

With respect to preliminary efficacy, due to attrition rates (i.e., 50% at baseline), an insufficient sample size was obtained to determine the intervention’s efficacy at improving girls’ body image and sports enjoyment. However, exploratory analyses were conducted on the mean scores of the primary and secondary outcomes, and the changes in mean scores were in the intended direction. That is, girls in the intervention condition reported improvements in body image and sports enjoyment, relative to the control condition who reported declines. A summary of these scores is reported in the Supplementary materials.

Based on the above findings, as well as consultation with stakeholders (i.e., girls, coaches, and community and industry partners), several modifications were made to the content and structure of BCA, which were deemed to increase intervention feasibility. This included reducing the number of sessions from five to three. That is, a three-session intervention was deemed more conducive to sport schedules and therefore would increase intervention adherence. Based on the intervention theory and aims (see Table 3), sessions one (Gender Stereotypes in Sport) and five (Advocacy in Sport) were removed, as these were considered more distal to girls’ body image and sports enjoyment, relative to the topics covered in the remaining three sessions. Further, the intervention content was reviewed for greater accessibility and inclusivity, particularly for girls with Physical Disabilities. Specifically, a Disability educator and activist with lived experience reviewed the intervention language and imagery, and changes were made to increase representation of girls with Physical Disabilities (e.g., adding examples into the intervention content that reflect their sport experience). Of note, this intervention is not yet suitable for all types of Disabilities (e.g., Deaf-Blindness; Hearing Impairments; Intellectual Disabilities, etc.), and therefore further adaptation and modifications are required before testing the intervention among girls living with these Disabilities.

With respect to the feasibility of conducting a two-armed, cluster randomised controlled trial in the US, the pilot highlighted several key limitations of the protocol, which required remedying before the main trial. First, the recruitment and onboarding period was lengthened from 3 to 6 months. This was based on feedback from coaches and their organisations that more time was needed to: 1) understand the research requirements; 2) get executive approval (if necessary) from the head of their organisation; 3) identify eligible participants; and 4) schedule the research tasks (i.e., surveys and intervention delivery). Further, due to the seasonal nature of some sports (i.e., soccer, lacrosse), this process became more complex for coaches, and therefore additional time was needed for troubleshooting. This modification aims to improve the onboarding of organisations, coaches, and girls, and subsequently reduce the attrition rates observed at baseline. Second, eligibility criteria were expanded to include school sports. Initially, only sport-based youth development organisations were targeted, as initial dissemination and implementation efforts were aimed at organisations that are typically underserved; however, due to the global COVID-19 pandemic, numerous organisations were experiencing funding and staffing cuts and therefore could not dedicate the time and resources to research participation. Therefore, the main trial is open to school athletic departments alongside youth development organisations, with the continued aim of targeting schools in underserved communities.

### Cluster randomised controlled (main) trial

#### Eligibility criteria

The current intervention has been developed for universal samples [42], in that it aims to provide population-wide benefits, irrespective of an individual’s level of risk or other relevant factors (e.g., type of sport, competition level). Therefore, increased risk for body image concerns or sports dropout are not inclusion criteria for this trial. Eligible participants will be girls aged 11–17 years old who play sport, English speaking, and US residents. Eligibility criteria will allow for different sport types (e.g., individual, team, aesthetic, non-aesthetic) and competition level (e.g., recreational, competitive). Coaches will be eligible to deliver the intervention, provided they currently occupy a coaching role (e.g., a coach at a school or sport recreational centre) and have a stable and ongoing relationship with the girls who will receive the intervention. Coaches and/or the organisations delivering the intervention will need to be available during both “Block A” (intervention condition) and “Block B” (waitlist control), with condition allocation masked from participants.

#### Measures

Research measures are presented in Table 4. One measure was purpose-built for the current research (i.e., girls’ comprehension of the intervention content). The remaining measures have been validated and widely used among adolescent girls, particularly in the US.

**Table 4:** Research outcomes and internal consistencies

Demographics	Age, gender identity, ethnicity, race, disability, state and region of residence, years playing sport	T1
Primary outcome		
Body Esteem	Body Esteem Scale for Adolescents & Adults (BESAA) [38] Appearance Esteem (AE; 10 items; e.g., I like how I look in photos) and Weight Esteem (WE; 8 items; e.g., I am satisfied with my weight) subscales Mean subscale scores range between 0 (never) and 4 (always). Higher scores reflect higher esteem The BESAA has shown good internal consistency reliability (Cronbach’s α = 0.76–0.96) and evidence of validity (correlations with self-esteem) in prior research conducted with adolescents [38, 43] The BESAA has been shown to detect change in body esteem at post-intervention [40, 44] and 1–6-month follow-up [40, 41, 45, 46] in interventions targeting adolescent girls	T1–T4
Secondary outcomes		
Body Appreciation	Body Appreciation Scale – 2 (BAS–2) [37] 10 items related to body appreciation (e.g., I take a positive attitude towards my body) Mean subscale scores range between 1 (never) and 5 (always). Higher scores reflect higher body appreciation The BAS–2 has shown good internal consistency reliability (Cronbach’s α = 0.86–0.93) and evidence of validity (correlations with self-esteem, body pride, life satisfaction) in prior research conducted with adolescents [46, 47] The BAS–2 has been shown to detect change in body appreciation at post-intervention [48] in interventions targeting adolescent girls	T1–T4
Resisting Objectification	Experience of Embodiment Scale (EES) – Resisting Objectification Subscale (RO) [49] 4 items related to inhabiting the body as a subjective site vs. as an objectified site (e.g., I focus more on what my body can do than on its appearance) Mean subscale scores range between 1 (strongly disagree) and 5 (strongly agree). Higher scores reflect greater resistance to objectification The RO subscale has shown good internal consistency reliability (Cronbach’s α = 0.71) and evidence of validity (correlations with body esteem, self-esteem) in previous research [49] The EES has been shown to detect change in embodiment at post-intervention and 3–12-month follow-up [17] in interventions targeting adolescent girls	T1–T4
Attuned Self-Care	Experience of Embodiment Scale (EES) – Attuned Self-Care Subscale (ASC) [49] 7 items related to the degree of attunement and responsiveness to the embodied self and its physical, emotional, relational, aspirational, and spiritual needs (e.g., I make sure I listen to my body and its needs [e.g., rest when I am tired, eat when hungry, leave when I feel unsafe, relax when stressed]) Mean subscale scores range between 1 (strongly disagree) and 5 (strongly agree). Higher scores reflect greater attunement to self-care The ASC subscale has shown good internal consistency reliability (Cronbach’s α = 0.75) and evidence of validity (correlations with body esteem, self-esteem) in previous research [49] The EES has been shown to detect change in embodiment at post-intervention and 3–12-month follow-up [17] in interventions targeting adolescent girls	T1–T4
Sports Enjoyment	Sources of Enjoyment in Youth Sport (SEYS) [39] 28 items related to six domains that elicit enjoyment among athletes (i.e., self-referenced competency [e.g., Playing well compared to how I’ve played in the past]; other-referenced competency and recognition [Doing skills other kids my age cannot do]; effort expenditure [Working hard in practice]; competitive excitement [Hearing the crowd cheer during a close game, match or race]; affiliation with peers [Being with friends on my team]; positive parental involvement [Getting encouragement from my parents]) Mean subscale scores range between 1 (strongly disagree) and 5 (strongly agree). Higher scores reflect greater enjoyment with this domain The SEYS has shown good internal consistency reliability (Cronbach’s α = 0.65–0.85) in previous research [39] The SEYS has been shown to detect change in enjoyment levels within domains at post-intervention in interventions targeting women [50] and differences in enjoyment among children and adolescents participating in individual and team sports [51]	T1–T4
Positive and Negative Affect	Positive and Negative Affect Scale (PANAS) [52] 10 items related to emotive states (5 positive [e.g., joyful]; 5 negative [e.g., scared]) Positive and negative affect subscale scores range between 1 (not at all) and 5 (extremely). Higher scores reflect greater positive or negative affect The PANAS has shown good internal consistency reliability (Cronbach’s α = 0.82–0.86) in previous research [52] The PANAS has been shown to detect change in affect at post-intervention [40, 53] in interventions targeting children and adolescents	T1–T4

T1 = Baseline; T2 = Post-Intervention; T3 = One-Month Follow-Up; T4 = Three-Month Follow-Up

#### Participant recruitment and procedure

A community sample of a minimum of 1,036 girls aged 11–17 years from diverse ethnic, geographic, and socio-economic backgrounds will be recruited for the main trial via a US recruitment partner who specialises in health education in school and community settings. Recruitment will occur in the 6 months prior to the trial start date (i.e., September 2022–February 2023) and will involve the following steps: 1) circulation of an online survey containing study information and an expression of interest and screening questionnaire (e.g., *Do you coach girls between 11–17 years old?*; *What is the estimated number of girls between 11–17 years that you will be coaching between February–July 2023?*) via the recruitment partner’s social media platforms and emailing lists; 2) following screening, eligible coaches and/or organisations will be invited to an information session held by the recruitment partner, where attendees will received a more detailed overview of the intervention and research tasks; and 3) provided coaches and/or organisations feel comfortable with, and confident that they can achieve, the research tasks, they will schedule an onboarding call with the third author (AT).

Following the onboarding process, organisations will be randomised into either the intervention (Block A) or waitlist control (Block B) condition (see Fig. 1 for participant flow). Those assigned to Block A will be advised to schedule the intervention between the 6^th^ February and 2^nd^ April 2023, while those assigned to Block B will be advised to schedule their delivery after the 2^nd^ July 2023. Three weeks prior to intervention delivery, coaches and parents will complete informed consent via an online survey. Following parental consent, girls in both conditions will complete informed assent and baseline assessments of primary and secondary outcomes via an online survey one week prior to intervention delivery. Girls will be encouraged to complete the surveys in the privacy of their homes; however, coaches agreed to make surveys accessible immediately before session one (e.g., girls will attend 20 min early and complete via a mobile device), should girls have difficulty completing the survey on their own accord. The intervention will be delivered in the team’s usual practice space, with logistics regarding equipment and space accessibility coordinated and confirmed with coaches during the onboarding call with the research team. At the end of the intervention period (i.e., 3 weeks), both conditions will be assessed on primary and secondary outcomes, and again at 1-month and 3-month follow-up. Following the final assessment, the waitlist control group will complete the intervention; however, their engagement will not be monitored or assessed. Lastly, to compensate participants for their time, they will receive an electronic voucher, with girls receiving US$60, coaches receiving $100, and organisations receiving $200.

#### Randomisation and blinding

Randomisation will occur at the group level (1:1). The randomisation sequence will be generated using a computer software, with clusters randomised into the intervention or control condition. Randomisation will be conducted by an experienced researcher who is external to the authoring research team and the conditions will be masked. Throughout the recruitment and onboarding processes, group allocation will be masked to participants. The intervention group will be referred to as Block A and the control condition referred to as Block B. Coaches and/or organisations delivering the intervention will be advised that two scheduling blocks have been created due to the magnitude of participants. Study hypotheses will also be masked from participants. Lastly, data analysts will be unaware of condition allocation (dummy coded) when conducting statistical analyses on primary and secondary outcomes.

#### Data monitoring and management

Based on previous evaluations of body image interventions among young people [40, 41], this intervention and trial protocol are deemed low risk for participant safety and therefore an independent data monitoring committee was not established. However, the research team will monitor survey responses for data quality, including completeness, consistency, and plausibility. All survey data, including informed consent and self-report measures, will be downloaded from a university approved survey software (Qualtrics), and securely stored in a password protected data file on a university approved cloud storage (i.e., OneDrive). At the start of each survey (e.g., T1, T2, T3, T4), participants will create a unique identification code, which will allow responses to be matched across time points. Personal data (i.e., name, geographical location, email address) of coaches, parents, and girls will be stored separately to survey data (i.e., outcome measures), with both datafiles securely stored in a password protected datafile on the university cloud storage. Personal data will be deleted once the trial is complete. Audio recordings will be used to assess intervention fidelity and will be securely stored on the university cloud server. Once the fidelity inter-rater reliability assessments are complete, these recordings will be permanently deleted. Anonymised survey data will be made available on reasonable request for non-commercial purposes.

#### Statistical power

A meta-analysis of 31 studies evaluating interventions that target the intersection of body image and movement experiences among girls and women via randomised controlled trials reported a mean effect size of Cohen’s *d* = 0.18 on body image, which can be interpreted as a small-to-medium effect size. Based on conservative *d* = 0.2, an ICC of 0.02 within intervention delivery groups (as informed by a similar cluster trial of a body image intervention Craddock, Budhraja, Garbett, Nasution, Gentili, Rizkiah, Saraswati, Medise, White, Diedrichs & Williamson: Randomised Control Trial of Dove Confident Me Indonesia: Single Session. Forthcoming]), and an approximate distribution of 15 athletes per delivery group, a total sample size of 864 athletes (*N* = 432 per arm) would provide 90% power over a correlation between baseline and outcome data at T2 or about 0.6, considering alpha = 0.05. We will oversample to mitigate the effects of loss at post-intervention due to athletes’ absence or any other logistical difficulty. Therefore, the ideal sample size will be increased by 20%, leading to a minimum target of approximately 1,036 athletes (518 per randomised arm).

#### Data analyses

Data preparation, assessment of baseline equivalence, hypothesis testing, and exploration will be undertaken using SPSS 28.0 (IBM Corp, 2021) and R Studio (R Core Team, 2021). Data will be screened to ensure scoring fidelity and data accuracy. Once the data validity checks are complete, scales’ scores will be calculated, score distributions will be assessed, and the data will be checked for outliers and any inaccurate influential observations. Missing data will be assessed across all outcomes and time points. Data will be analysed on an intention-to-treat basis and secondly using data from participants with at least 70% of the attention checks successful completed.

The primary analyses will be linear mixed models with random intercepts for all outcome measures (body esteem, body appreciation, self-objectification, attuned self-care, sports enjoyment, and affect). The models will include two main effects: study phase (i.e., repeated measures fixed effect factor with three levels: T2, T3, and T4), randomised arm (i.e., between-subjects fixed effect factor with two levels: intervention and control), as well as baseline levels of the outcome as a covariate. The model will also see two two-way interactions (i.e., randomised arm by study phase, randomised arm by baseline). Analyses will be run on an intention-to-treat basis, both with and without clusters including a random effect (i.e., intervention delivery group), in order to identify their potential significant impact [54].

For each outcome measure, a priori ANCOVAs will compare the two randomised arms at T2, controlling for baseline measures as covariates. These analyses will be repeated at T3 and T4. If the homogeneity of regression lines assumption is violated, the ANCOVA model will include a baseline by randomised arm interaction. Exploratory moderation and mediation analyses will be run to check whether any differences in intervention efficacy might be dependent on relevant demographic or baseline variables. To ensure valid statistical inferences, we will check for any potential assumption violation in all the models.

#### Ethical issues, approval, and trial registration

This research adheres to the ethical standards and guidelines put forth by the University of Minnesota and the University of the West of England. Specifically, participants will receive an overview of project aims, objectives, and research tasks prior to providing consent. Further, participation is voluntary, and participants may withdraw at any stage without justification or penalty. Irrespective of their completion stage, all participants will receive a debrief form that discloses the research aims, along with additional mental health resources (e.g., evidence-base websites, helpline contact details). Any serious adverse events will be reported to the universities’ ethics committees. This study was reviewed by and received approval from the ethics committees at the University of Minnesota (STUDY00012457) and the University of the West of England (HAS.21.03.120c). The study is registered with Clinical Trials.Gov (https://www.clinicaltrials.gov/ct2/show/NCT05594524).

#### Dissemination

In accordance with the broader aims of this research, which are grounded in CBPR principles, findings from this research will be widely disseminated beyond the academic setting (e.g., peer-reviewed publication and academic conferences). Research findings, as well as the intervention materials will be hosted on a freely accessible website, as well as promoted via promotional campaigns. If BCA proves effective, it will be scaled up and delivered to young people across the US and other English-speaking countries, with future plans including the translation and localisation of the programme for non-English speaking countries (e.g., France, India, Mexico).

## Discussion

This protocol reports on the co-creation, piloting, and planned cluster randomised controlled trial of a new intervention, Body Confident Athletes (BCA). This coach-led intervention aims to increase girls’ positive body image and sports enjoyment. An initial pilot assessed intervention feasibility, as well as the feasibility of conducting a two-armed cluster randomised controlled trial in the US. Findings from the pilot led to modifications to the intervention content (i.e., greater representation of girls with Disabilities in sport) and structure (i.e., reduced the core sessions from five to three), as well as modifications to the trial protocol (i.e., expanding the eligibility criteria and recruitment channels to include school athletic departments). Based on these amendments, the intervention and trials were deemed feasible with the main trial commencing 30 January 2023 (i.e., first wave of baseline assessments) and recruitment finishing 22 February 2023 (i.e., last opportunity to enrol). The main trial will assess the immediate (post-intervention) and short-term (1-month, 3-month) impact of BCA on girls’ body image (body esteem, body appreciation, attuned self-care, resisting objectification), sports enjoyment, and affect (positive and negative). At the completion of the trial, provided BCA is effective and does not require major revisions (e.g., is found ineffective or harmful), the intervention materials will be made freely available via an English website in October 2023.

This research makes several advancements in addressing the intersection of girls’ body image and sport experiences. To our knowledge, this is the first intervention on this topic to: 1) utilise a global and cross-national lens and community-based participatory research (CBPR) throughout the intervention development process; 2) target both risk and protective factors for body image and apply intervention techniques to sport-specific settings and scenarios; and 3) task shift intervention delivery to coaches. Relatedly, and to our knowledge, it is the first study on this topic to pilot test the feasibility of both the intervention and trial protocol prior to moving into a large-scale cluster randomised controlled trial. Collectively, these theoretical and methodological considerations allowed us to incrementally refine both the intervention (i.e., content, structure, and format) and the trial protocol (i.e., eligibility criteria and recruitment pathways). In turn, the main trial will be conducted on the most feasible intervention and under the most feasible conditions. If proven effective at enhancing girls’ positive body image and sports enjoyment in the US, BCA will be tested for effectiveness in other countries and cultures. Overall, these research efforts seek to increase BCA’s potential at improving girls’ positive body image and sports enjoyment across the world, which may possibly help with reducing genders disparities in sports participation.

## Supplementary Information

**Additional file 1:** **Supplementary Figure 1.** The pilot research design according to the Consolidated Standards of Reporting Trials (CONSORT). **Supplementary Table 1.** SPIRIT 2013 Checklist: Recommended items to address in a clinical trial protocol and related documents. **Supplementary Table 2.** CONSORT 2010 Checklist and Extension Items for Cluster Randomised Trials. **Supplementary Table 3.** Coaches’ Demographic and Acceptability Scores for Intervention Pilot. **Supplementary Table 4.** Girls’ Demographics for the Intervention Pilot. **Supplementary Table 5.** Girls’ Efficacy Data for the Intervention Pilot. **Supplementary Table 6.** Girls’ Acceptability and Attendance Data for the Intervention Pilot. Sample of Girls’ Questionnaire administered at Baseline (T1), Post-Intervention (T2), 1-month (T3) and 3-Month (T4) Follow-Up.

## Data Availability

The intervention, Body Confident Athletes, will be freely available via a website that will be launched in October 2023. The datasets generated and/or analysed during the current study are not publicly available as per the data management and monitoring guidelines of the governing universities but are available from the corresponding author on reasonable request.

## Abbreviations

AE: Appearance Esteem
BCA: Body Confident Athletes
CBPR: Community-Based Participatory Research
CONSORT: Consolidated Standards of Reporting Trials of Electronic Health
HBI: Healthy Body Image
SPIRIT: Standard Protocol Items: Recommendations for Interventional Trials
T1, T2, T3, T4: Time One, Time Two, Time Three, Time Four
UK: United Kingdom
US: United States
WE: Weight Esteem
