# Supplementary material for: The co-creation, initial piloting, and protocol for a cluster randomised controlled trial of a coach-led positive body image intervention for girls in sport
Source: BMC Public Health. 2023 Jul 31;23:1467. doi: 10.1186/s12889-023-16360-w (PMC10391850; doi:10.1186/s12889-023-16360-w)
Supplement: Supplementary file 1 — Additional file 1: Supplementary Figure 1. The pilot research design according to the Consolidated Standards of Reporting Trials (CONSORT). Supplementary Table 1. SPIRIT 2013 Checklist: Recommended items to address in a clinical trial protocol and related documents. Supplementary Table 2. CONSORT 2010 Checklist and Extension Items for Cluster Randomised Trials. Supplementary Table 3. Coaches’ Demographic and Acceptability Scores for Intervention Pilot. Supplementary Table 4. Girls’ Demographics for the Intervention Pilot. Supplementary Table 5. Girls’ Efficacy Data for the Intervention Pilot. Supplementary Table 6. Girls’ Acceptability and Attendance Data for the Intervention Pilot. Sample of Girls’ Questionnaire administered at Baseline (T1), Post-Intervention (T2), 1-month (T3) and 3-Month (T4) Follow-Up. [file 12889_2023_16360_MOESM1_ESM.docx]

Supplementary Materials

Excluded (*k* = 10):

Declined to participate (*k* = 1)

Did not meet inclusion criteria (*k* = 2)

Did not respond to initial call (*k* = 7)

Sports organisations randomised (*k* = 8)

Sports organisations screened for eligibility

(*k* = 18)

**Enrolment**

**Allocation**

Organisations allocated to intervention (*k* = 4)

- Received allocated intervention (*k* = 2; athletes *n* = 13)
- Did not receive allocated intervention (*k* = 2; stopped responding to communication efforts)

Organisations allocated to waitlist control (*k* = 4)

- Received allocated intervention (k = 2; athletes n = 13)
- Did not receive allocated intervention
- (k = 2; stopped responding to communication efforts)

**Baseline**

Athletes completed baseline assessments (*n* = 9)

- **Demographics:** age, ethnicity, sport experience
- **Target outcomes:** trait body image, sports enjoyment

00

Athletes completed baseline assessments (*n* = 10)

- **Demographics:** age, ethnicity, sport experience
- **Target outcomes:** trait body image, sports enjoyment

Standard care (no intervention)

**Intervention**

Athletes took part in the **Body Confident Athletes** intervention (five x 60-minute sessions led by their coach over five consecutive weeks)

Athletes completed post-intervention assessments (*n* = 5)

- **Target outcomes:** trait body image, sports enjoyment

**Post-Intervention**

Athletes completed post-intervention assessments (*n* = 7)

- **Target outcomes:** trait body image, sports enjoyment

**Acceptability outcomes**

Analysed (*n* = 9)

**Analysis**

Analysed (*n* = 10)

Supplementary Figure 1. The pilot research design according to the Consolidated Standards of Reporting Trials (CONSORT)

Supplementary Table 1

SPIRIT 2013 Checklist: Recommended items to address in a clinical trial protocol and related documents

| **Section/item** | **Item No** | **Description** | **Page Ref** |
| --- | --- | --- | --- |
| **Administrative information** | | | |
| Title | 1 | Descriptive title identifying the study design, population, interventions, and, if applicable, trial acronym | 1 |
| Trial registration | 2a | Trial identifier and registry name. If not yet registered, name of intended registry | 2, 18 |
|  | 2b | All items from the World Health Organization Trial Registration Data Set | N/A |
| Protocol version | 3 | Date and version identifier | 1 |
| Funding | 4 | Sources and types of financial, material, and other support | 21 |
| Roles and responsibilities | 5a | Names, affiliations, and roles of protocol contributors | 1, 21 |
|  | 5b | Name and contact information for the trial sponsor | 1 |
|  | 5c | Role of study sponsor and funders, if any, in study design; collection, management, analysis, and interpretation of data; writing of the report; and the decision to submit the report for publication, including whether they will have ultimate authority over any of these activities | 21 |
|  | 5d | Composition, roles, and responsibilities of the coordinating centre, steering committee, endpoint adjudication committee, data management team, and other individuals or groups overseeing the trial, if applicable (see Item 21a for data monitoring committee) | N/A |

| **Introduction** | | | |
| --- | --- | --- | --- |
| Background and rationale | 6a | Description of research question and justification for undertaking the trial, including summary of relevant studies (published and unpublished) examining benefits and harms for each intervention | 3-7 |
|  | 6b | Explanation for choice of comparators | 6 |
| Objectives | 7 | Specific objectives or hypotheses | 6-7 |
| Trial design | 8 | Description of trial design including type of trial (e.g., parallel group, crossover, factorial, single group), allocation ratio, and framework (e.g., superiority, equivalence, noninferiority, exploratory) | 6-7, 29 |
| **Methods: Participants, interventions, and outcomes** | | | |
| Study setting | 9 | Description of study settings (e.g., community clinic, academic hospital) and list of countries where data will be collected. Reference to where list of study sites can be obtained | 7-8, 14-15 |
| Eligibility criteria | 10 | Inclusion and exclusion criteria for participants. If applicable, eligibility criteria for study centres and individuals who will perform the interventions (e.g., surgeons, psychotherapists) | 7-8 |
| Interventions | 11a | Interventions for each group with sufficient detail to allow replication, including how and when they will be administered | 8-9, 14-15, 38-42 |
|  | 11b | Criteria for discontinuing or modifying allocated interventions for a given trial participant (e.g., drug dose change in response to harms, participant request, or improving/worsening disease) | N/A |
|  | 11c | Strategies to improve adherence to intervention protocols, and any procedures for monitoring adherence (e.g., drug tablet return, laboratory tests) | 13 |
|  | 11d | Relevant concomitant care and interventions that are permitted or prohibited during the trial | N/A |
| Outcomes | 12 | Primary, secondary, and other outcomes, including the specific measurement variable (e.g., systolic blood pressure), analysis metric (e.g., change from baseline, final value, time to event), method of aggregation (e.g., median, proportion), and time point for each outcome. Explanation of the clinical relevance of chosen efficacy and harm outcomes is strongly recommended | 2, 6-7, 30-31, 43-44 |
| Participant timeline | 13 | Time schedule of enrolment, interventions (including any run-ins and washouts), assessments, and visits for participants. A schematic diagram is highly recommended (see Figure) | 30-31 |
| Sample size | 14 | Estimated number of participants needed to achieve study objectives and how it was determined, including clinical and statistical assumptions supporting any sample size calculations | 16-17 |
| Recruitment | 15 | Strategies for achieving adequate participant enrolment to reach target sample size | 13-15 |
| **Methods: Assignment of interventions (for controlled trials)** | | | |
| Allocation: |  |  |  |
| Sequence generation | 16a | Method of generating the allocation sequence (e.g., computer-generated random numbers), and list of any factors for stratification. To reduce predictability of a random sequence, details of any planned restriction (e.g., blocking) should be provided in a separate document that is unavailable to those who enrol participants or assign interventions | 15 |
| Allocation concealment mechanism | 16b | Mechanism of implementing the allocation sequence (e.g., central telephone; sequentially numbered, opaque, sealed envelopes), describing any steps to conceal the sequence until interventions are assigned | 8, 15 |
| Implementation | 16c | Who will generate the allocation sequence, who will enrol participants, and who will assign participants to interventions | 15 |
| Blinding (masking) | 17a | Who will be blinded after assignment to interventions (e.g., trial participants, care providers, outcome assessors, data analysts), and how | 15 |
|  | 17b | If blinded, circumstances under which unblinding is permissible, and procedure for revealing a participant’s allocated intervention during the trial | 15 |
| **Methods: Data collection, management, and analysis** | | | |
| Data collection methods | 18a | Plans for assessment and collection of outcome, baseline, and other trial data, including any related processes to promote data quality (e.g., duplicate measurements, training of assessors) and a description of study instruments (e.g., questionnaires, laboratory tests) along with their reliability and validity, if known. Reference to where data collection forms can be found, if not in the protocol | 14-15, 43-44 |
|  | 18b | Plans to promote participant retention and complete follow-up, including list of any outcome data to be collected for participants who discontinue or deviate from intervention protocols | 14-15 |
| Data management | 19 | Plans for data entry, coding, security, and storage, including any related processes to promote data quality (e.g., double data entry; range checks for data values). Reference to where details of data management procedures can be found, if not in the protocol | 15-16 |
| Statistical methods | 20a | Statistical methods for analysing primary and secondary outcomes. Reference to where other details of the statistical analysis plan can be found, if not in the protocol | 16-17 |
|  | 20b | Methods for any additional analyses (e.g., subgroup and adjusted analyses) | 17 |
|  | 20c | Definition of analysis population relating to protocol non-adherence (e.g., as randomised analysis), and any statistical methods to handle missing data (e.g., multiple imputation) | 16-17 |
| **Methods: Monitoring** | | | |
| Data monitoring | 21a | Composition of data monitoring committee (DMC); summary of its role and reporting structure; statement of whether it is independent from the sponsor and competing interests; and reference to where further details about its charter can be found, if not in the protocol. Alternatively, an explanation of why a DMC is not needed | 15-16 |
|  | 21b | Description of any interim analyses and stopping guidelines, including who will have access to these interim results and make the final decision to terminate the trial | N/A |
| Harms | 22 | Plans for collecting, assessing, reporting, and managing solicited and spontaneously reported adverse events and other unintended effects of trial interventions or trial conduct | 15-16 |
| Auditing | 23 | Frequency and procedures for auditing trial conduct, if any, and whether the process will be independent from investigators and the sponsor | - |
| **Ethics and dissemination** | | | |
| Research ethics approval | 24 | Plans for seeking research ethics committee/institutional review board (REC/IRB) approval | 17-18, 20 |
| Protocol amendments | 25 | Plans for communicating important protocol modifications (e.g., changes to eligibility criteria, outcomes, analyses) to relevant parties (e.g., investigators, REC/IRBs, trial participants, trial registries, journals, regulators) | - |
| Consent or assent | 26a | Who will obtain informed consent or assent from potential trial participants or authorised surrogates, and how (see Item 32) | 14-15 |
|  | 26b | Additional consent provisions for collection and use of participant data and biological specimens in ancillary studies, if applicable | N/A |
| Confidentiality | 27 | How personal information about potential and enrolled participants will be collected, shared, and maintained in order to protect confidentiality before, during, and after the trial | 15-16 |
| Declaration of interests | 28 | Financial and other competing interests for principal investigators for the overall trial and each study site | 20-21 |
| Access to data | 29 | Statement of who will have access to the final trial dataset, and disclosure of contractual agreements that limit such access for investigators | 15-16 |
| Ancillary and post-trial care | 30 | Provisions, if any, for ancillary and post-trial care, and for compensation to those who suffer harm from trial participation | 15, 17 |
| Dissemination policy | 31a | Plans for investigators and sponsor to communicate trial results to participants, healthcare professionals, the public, and other relevant groups (e.g., via publication, reporting in results databases, or other data sharing arrangements), including any publication restrictions | 18 |
|  | 31b | Authorship eligibility guidelines and any intended use of professional writers | 21 |
|  | 31c | Plans, if any, for granting public access to the full protocol, participant-level dataset, and statistical code | 16 |
| **Appendices** | | | |
| Informed consent materials | 32 | Model consent form and other related documentation given to participants and authorised surrogates | Supplementary  materials |
| Biological specimens | 33 | Plans for collection, laboratory evaluation, and storage of biological specimens for genetic or molecular analysis in the current trial and for future use in ancillary studies, if applicable | N/A |

Supplementary Table 2

CONSORT 2010 Checklist and Extension Items for Cluster Randomised Trials

| Section | CONSORT 2010 Item | | Addressed on Page No | CONSORT-Cluster Extension | | Addressed on Page No |
| --- | --- | --- | --- | --- | --- | --- |
| **Title and Abstract** | | | | | | |
| **Title and Abstract** | 1a | Identification as a randomised trial in the title | 1 | 1a | Identification as a cluster randomised trial in the title | 1 |
|  | 1b | Structured summary of trial design, methods, results, and conclusions (for specific guidance see CONSORT for abstracts) | 2 | 1b |  |  |
| **Introduction** | | | | | | |
| **Background and objectives** | 2a | Scientific background and explanation of rationale | 3-7 | 2a | Rationale for using a cluster design | 6 |
|  | 2b | Specific objectives or hypotheses | 6-7 | 2b | Whether objectives pertain to the cluster level, the individual participant level or both | 7 |
| **Method** | | | | | | |
| **Trial design** | 3a | Description of trial design (such as parallel, factorial) including allocation ratio | 6-7 | 3a | Definition of cluster and description of how the design features apply to the clusters |  |
|  | 3b | Important changes to methods after trial commencement (such as eligibility criteria), with reasons | N/A | 3b |  |  |
| **Participants** | 4a | Eligibility criteria for participants | 8 | 4a | Eligibility criteria for clusters | 8 |
|  | 4b | Settings and locations where the data were collected | 7-8, 14-15 | 4b |  |  |
| **Interventions** | 5 | The interventions for each group with sufficient details to allow replication, including how and when they were actually administered | 8-9, 14-15, 38-42 | 5 | Whether interventions pertain to the cluster level, the individual participant level or both | 8-9, 14-15, 38-42 |
| **Outcomes** | 6a | Completely defined pre-specified primary and secondary outcome measures, including how and when they were assessed | 2, 6-7, 30-31, 43-44 | 6a | Whether outcome measures pertain to the cluster level, the individual participant level or both | 2, 6-7, 30-31, 43-44 |
|  | 6b | Any changes to trial outcomes after the trial commenced, with reasons | N/A | 6b |  |  |
| **Sample size** | 7a | How sample size was determined | 16 | 7a | Method of calculation, number of clusters(s) (and whether equal or unequal cluster sizes are assumed), cluster size, a coefficient of intracluster correlation (ICC or k), and an indication of its uncertainty | 16 |
|  | 7b | When applicable, explanation of any interim analyses and stopping guidelines | N/A | 7b |  |  |
| **Randomisation** | | | | | | |
| **Sequence generation** | 8a | Method used to generate the random allocation sequence | 15 | 8a |  |  |
|  | 8b | Type of randomisation; details of any restriction (such as blocking and block size) | 15 | 8b | Details of stratification or matching if used | 15 |
| **Allocation concealment mechanism** | 9 | Mechanism used to implement the random allocation sequence (such as sequentially numbered containers), describing any steps taken to conceal the sequence until interventions were assigned | 15 | 9 | Specification that allocation was based on clusters rather than individuals and whether allocation concealment (if any) was at the cluster level, the individual participant level or both | 15 |

| **Implementation** | 10 | Who generated the random allocation sequence, who enrolled participants, and who assigned participants to interventions | 15 | 10 | Replace by 10a, 10b and 10c |  |
| --- | --- | --- | --- | --- | --- | --- |
|  |  |  |  | 10a | Who generated the random allocation sequence, who enrolled clusters, and who assigned clusters to interventions |  |
|  |  |  |  | 10b | Mechanism by which individual participants were included in clusters for the purposes of the trial (such as complete enumeration, random sampling) |  |
|  |  |  |  | 10c | From whom consent was sought (representatives of the cluster, or individual cluster members, or both), and whether consent was sought before or after randomisation |  |
| **Blinding** | 11a | If done, who was blinded after assignment to interventions (for example, participants, care providers, those assessing outcomes) and how | 15 | 11a |  |  |
|  | 11b | If relevant, description of the similarity of interventions | N/A | 11b |  |  |
| **Statistical methods** | 12a | Statistical methods used to compare groups for primary and secondary outcomes | 16-17 | 12a | How clustering was taken into account | 16-17 |
|  | 12b | Methods for additional analyses, such as subgroup analyses and adjusted analyses | 17 | 12b |  |  |
| **Results** | | | | | | |
| **Participant flow** (a diagram is strongly recommended) | 13a | For each group, the numbers of participants who were randomly assigned, received intended treatment, and were analysed for the primary outcome | N/A | 13a | For each group, the numbers of clusters that were randomly assigned, received intended treatment, and were analysed for the primary outcome | N/A |
|  | 13b | For each group, losses and exclusions after randomisation, together with reasons | N/A | 13b | For each group, losses and exclusions for both clusters and individual cluster members | N/A |
| **Recruitment** | 14a | Dates defining the periods of recruitment and follow-up | 31-32 | 14a |  |  |
|  | 14b | Why the trial ended or was stopped | N/A | 14b |  |  |
| **Baseline data** | 15 | A table showing baseline demographic and clinical characteristics for each group | N/A | 15 | Baseline characteristics for the individual and cluster levels as applicable for each group | N/A |
| **Numbers analysed** | 16 | For each group, number of participants (denominator) included in each analysis and whether the analysis was by original assigned groups | N/A | 16 | For each group, number of clusters included in each analysis | N/A |
| **Outcomes and estimation** | 17a | For each primary and secondary outcome, results for each group, and the estimated effect size and its precision (such as 95% confidence interval) | N/A | 17a | Results at the individual or cluster level as applicable and a coefficient of intracluster correlation (ICC or k) for each primary outcome | N/A |
|  | 17b | For binary outcomes, presentation of both absolute and relative effect sizes is recommended | N/A | 17b |  |  |
| **Ancillary analyses** | 18 | Results of any other analyses performed, including subgroup analyses and adjusted analyses, distinguishing pre-specified from exploratory | N/A | 18 |  |  |
| **Harms** | 19 | All important harms or unintended effects in each group (for specific guidance see CONSORT for harms) | N/A | 19 |  |  |

| **Discussion** | | | | | | |
| --- | --- | --- | --- | --- | --- | --- |
| **Limitations** | 20 | Trial limitations, addressing sources of potential bias, imprecision, and, if relevant, multiplicity of analyses | N/A | 20 |  |  |
| **Generalisability** | 21 | Generalisability (external validity, applicability) of the trial findings | N/A | 21 | Generalisability to clusters and/or individual participants (as relevant) | N/A |
| **Interpretation** | 22 | Interpretation consistent with results, balancing benefits and harms, and considering other relevant evidence | N/A | 22 |  |  |
| **Other Information** | | | | | | |
| **Registration** | 23 | Registration number and name of trial registry | 2, 18 | 23 |  |  |
| **Protocol** | 24 | Where the full trial protocol can be accessed, if available | N/A | 24 |  |  |
| **Funding** | 25 | Sources of funding and other support (such as supply of drugs), role of funders | 22 | 25 |  |  |

Supplementary Table 3

Coaches’ Demographic and Acceptability Scores for Intervention Pilot

|  | **Total (N = 7)** | **Intervention (N = 4)** | **Control (N = 3)** |
| --- | --- | --- | --- |
| Age M(SD) | 29.71 (9.16) | 28.25 (7.32) | 31.66 (12.70) |
| Gender N(%) |  |  |  |
| Female | 7 (100%) | 4 (100%) | 3 (100%) |
| Ethnicity N(%) |  |  |  |
| Black or African American | 3 (42.85%) | 1 (25%) | 2 (66.67%) |
| White | 3 (42.85%) | 2 (50%) | 1 (33.34%) |
| Multiracial/Biracial | 1 (14.28%) | 1 (25%) | - |
| Geolocation |  |  |  |
| Amarillo | 1 (14.28%) | - | 1 (33.34%) |
| Atlanta Ga | 1 (14.28%) | 1 (25%) | - |
| Chicago | 1 (14.28%) | - | 1 (33.34%) |
| Detroit | 1 (14.28%) | 1 (25%) | - |
| New Orleans | 1 (14.28%) | - | 1 (33.34%) |
| San Francisco | 2 (28.57%) | 2 (50%) | - |
| Acceptability & Feasibility of Materials | Score Range | M(SD) |  |
| Material Topics | 1-5 | 4.21 (0.87) |  |
| Structural Integration | 1-5 | 4.00 (0.62) |  |

*Note*. Acceptability and feasibility scores are based on 3 coaches’ responses.

Supplementary Table 4

Girls’ Demographics for the Intervention Pilot

|  | **Total (N = 26)** | **Intervention (N = 13)** | **Control (N = 13)** |
| --- | --- | --- | --- |
| Age M(SD) | 12.73 (1.99) | 12.92 (1.85) | 12.54 (2.18) |
| Gender N(%) |  |  |  |
| Female | 25 (96.15%) | 12 (92.31%) | 13 (100%) |
| Prefer to self-describe | 1 (3.84%) | 1 (7.69%) | - |
| Ethnicity N(%) |  |  |  |
| Asian | 3 (11.54%) | 3 (23.07%) | - |
| Black or African American | 5 (19.23%) | - | 5 (38.46%) |
| Hispanic, Latino/a, or Spanish origin | 6 (23.07%) | 1 (7.69%) | 5 (38.46%) |
| White | 9 (34.61%) | 6 (46.15%) | 3 (23.07%) |
| Multiracial/Biracial | 2 (7.69%) | 2 (15.38%) | - |
| Prefer not to say | 1 (3.84%) | 1 (7.69%) | - |

Supplementary Table 5

Girls’ Efficacy Data for the Intervention Pilot

|  | **Intervention** | | **% change** | **Control** | | **% change** |
| --- | --- | --- | --- | --- | --- | --- |
|  | **T1** | **T2** |  | **T1** | **T2** |  |
| Body Esteem (0-4) | 2.34 (0.95) | 2.99 (0.84) | +16.25‬% | 2.19 (0.56) | 1.87 (0.63) | -8% |
| Appearance esteem | 2.35 (0.89) | 3 (0.73) | +16.25‬% | 2.2 (0.60) | 1.86 (0.77) | -8.5% |
| Weight esteem | 2.34 (1.08) | 2.98 (0.99) | +16% | 2.18 (0.56) | 1.90 (0.47) | -7‬% |
| Sports Enjoyment (1-5) | 3.72 (0.37) | 3.96 (0.35) | +4.8‬% | 3.88 (0.47) | 3.46 (0.47) | -8.4‬% |
| Self-referenced competency | 4.2 (0.57) | 4.36 (0.52) | +3.2‬% | 4.14 (0.80) | 3.5 (0.47) | -12.8% |
| Effort expenditure | 3.78 (0.68) | 4.03 (0.60) | +5% | 4.12 (0.81) | 3.56(0.41) | -11.2% |
| Other-referenced competency and recognition | 2.94 (0.69) | 3.21 (0.38) | +5.4‬% | 3.46 (0.63) | 3.24 (0.48) | -4.4‬% |
| Affiliation with peers | 4.08 (0.57) | 4.26 (0.64) | +3.6‬% | 3.93 (0.84) | 3.6 (0.47) | -6.6‬% |
| Competitive excitement | 3.92 (0.55) | 4.07 (0.64) | +3% | 3.83 (0.66) | 3.4 (0.65) | -8.6% |
| Positive parental involvement | 3.72 (0.64) | 4.11 (0.47) | +7.8‬% | 3.95 (0.74) | 3.5 (0.70) | -9.2% |

Supplementary Table 6

Girls’ Acceptability and Attendance Data for the Intervention Pilot

| Acceptability of Materials  M(SD) (1-5) | 4.18 (0.38) |
| --- | --- |
| Attendance N(%) |  |
| Session 1 | 7 (53.84%) |
| Session 2 | 7 (53.84%) |
| Session 3 | 4 (30.77%) |
| Session 4 | 3 (23.07%) |
| Session 5 | 6 (46.15%) |
| Attended All Sessions | 2 (15.38%) |

**Coaches’ Consent**


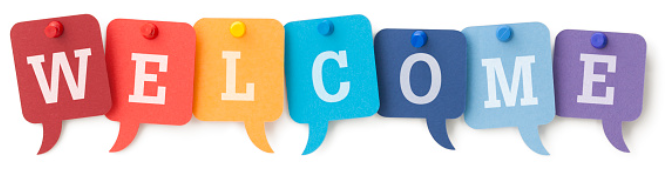


You have received this survey because your sports club or school has suggested that you might like to participate in our research study.

In the first part of the survey you will be provided with information about the research we are conducting on the ***Body Confident Athletes*** program. You will then indicate whether you consent to participate.

If you decide to participate in this research, you will move on to the second part of the survey, which will include a series of demographic questions.

You do not have to participate in this research. It is voluntary and you can withdraw at any time.

Completing part one and two of this survey should take no longer than 10 minutes of your time. Please make sure you complete the whole survey.

[Page Break]

**Part One - Research Information**

**Information About This Study (page 1 of 4)**

**Title of Research Study:**Developing Embodied Programs for Girls and Coaches

**Investigator Team Contact Information:** Dr Courtney Boucher, Dr Emily Matheson

For questions about research appointments, the research study, research results, or other concerns, contact the study team at:

| **Researcher Name** | Dr Courtney Boucher | **Researcher Name** | Dr Emily Matheson |
| --- | --- | --- | --- |
| **Researcher Affiliation** | Tucker Center for Research on Girls & Women in Sport, University of Minnesota | **Researcher Affiliation** | Centre for Appearance Research, University of the West of England |
| **Email Address** | bouch114@umn.edu | **Email Address** | emily.matheson@uwe.ac.uk |

**Supported By:**Centre for Appearance Research, Tucker Center for Research on Girls & Women in Sport

**Key Information About This Research Study**

The following is a short summary to help you decide whether or not to be a part of this research study. More detailed information is listed later on in this survey.

**What is research?**
Doctors and researchers are committed to your care and safety. There are important differences between research and treatment plans:

- The goal of research is to learn new things in order to help groups of people in the future. Researchers learn things by following the same plan with a number of participants, so they do not usually make changes to the plan for individual research participants. You, as an individual, may or may not be helped by volunteering for a research study.
- The goal of treatment is to help you get better or to improve your quality of life. Doctors can make changes to your treatment plan as needed.

**Why am I being invited to take part in this research study?**
We are asking you to take part in this research study because you are currently coaching a girls’ sports team. This information form will provide an overview of the research.

**What should I know about being in a research study?**
Someone will explain this research study to you. Whether or not you take part is up to you. You can choose not to take part. You can agree to take part and later change your mind. Your decision will not be held against you. You can ask all the questions you want before you decide.

**Why is this research being done?**
Girls often worry about their appearance when playing sport, which can reduce their enjoyment and performance in sport. These worries are worsened by athletic appearance ideals promoted by society, ill-fitting and objectifying uniforms for girls and women, and appearance-related teasing from peers and coaches.

Currently, there is no rigorously tested, effective, or scalable program that is delivered by coaches and improves girls’ body image and sports enjoyment. We have designed the ***Body Confident Athletes*** program for girls, which aims to:

- Stop girls from dropping out of sport because of body image concerns.
- Help girls reclaim confidence in their bodies within sporting contexts.

**How long will the research last?**
We expect that your participation in this research study will last 3 weeks. You will complete a brief online training in the program and deliver a 3 week in-person program to girls. The sessions last one hour and we will aim to schedule these within your regular activities at your sports club or school.

**What will I need to do to participate?**
With your consent, you will deliver a body image program at your sports club or school. The program will include 3 in-person sessions and will be led by you. More detailed information about the study procedures can be found under the section**“What happens if I say “Yes, I want to be in this research”?”**.

**Is there any way being in this study could be bad for me?**
Based on our research to date, we fully expect that this research will positively impact you. We will be talking about sensitive topics, such as how you and the girls think and feel about your bodies. However, it is your choice to share as little or as much as you feel comfortable with during the sessions.

**Will being in this study help me in any way?**
You will play an integral role in developing a program that helps girls and coaches address body image concerns within sport settings.

**What happens if I do not want to be in this research?**
You may decline to participate and it will not be held against you.

[Page Break]

**Information About This Study (page 2 of 4)**

**Detailed Information About This Research Study**

The following is more detailed information about this study in addition to the information listed on the previous page.

**How many people will be studied?**
We expect about 200 coaches and 2,000 girls will participate in this research study.

**What happens if I say “Yes, I want to be in this research”?**
With your consent, you will deliver a body image program at your sports club or school. The program will include 3 in-person sessions and will be led by you. A group of girls and coaches from your club or school will be asked to:

- Participate in a 3 week in-person program that is delivered by the coaches.
- Activities will include group discussions and physical activities, and individual writing and reflection activities aimed at improving girls’ body image.
- Sometimes, we may record the sessions. This is to make sure the program is being delivered as planned. Recordings will not be shared with anyone outside of the research team.
- Girls will complete four online surveys over the course of the research project, including one before the program starts, and three after the program finishes.
- Coaches will complete an online survey before and after they participate in the program.
- These questionnaires will be anonymized, and will not be shared or read by anyone, other than the small research team.
- During the program and when answering the online surveys, there are no right or wrong answers.
- What you share during this research will be confidential. That is, we will not share what you say during the sessions or in the questionnaires. On rare occasions, we may need to share your responses, if someone is worried about your or someone else’s safety.
- In recognition of your contribution to this research, you will receive a small monetary compensation for your time. You will receive up to $100 dollars at the end of the study (i.e., after completing the second online survey).

**What data do we collect?**
The online surveys comprise of demographic questions and self-report measures about your coaching experience. These measures have been validated and peer-reviewed for use among young people and adults. To date, no adverse effects have been reported among participants following exposure to questionnaires of this kind.

**How long do we keep your data for?**
All forms of raw data will be stored for as long as they retain research value. After that time all raw data will be destroyed; however, the project supervisor will retain a computer version of the dataset (e.g., transcriptions, questionnaire responses), which will be stored in password-protected files on a secure server at all times for up to two years after study completion.

Only the core research team will have access to the recordings and questionnaire responses. These data will be shared with co-investigators and partners (e.g., via Microsoft Teams Share Screen), but they will not retain the data.

All data sets will be password protected, with access granted only to the primary researchers on the project. In the event that data needs to be shared with external collaborators (e.g., the research funders), then it will be shared using University of Minnesota approved methods. All collaborators will also agree to the data management terms for this project.

An optional element will be added to collect email addresses to contact participants about future research opportunities. Email addresses will be stored separately from research data and will not be used to identify participant responses.

[Page Break]

**Information About This Study (page 3 of 4)**

**What happens if I say “Yes”, but I change my mind later?**
Choosing not to be in this study or to stop being in this study will not result in any penalty to you or loss of benefit to which you are entitled. At any time, you may decide to withdraw from the study. If you withdraw, no more information will be collected from you. When you indicate that you wish to withdraw, the information already collected will be used in the study unless you specify that you would like your data destroyed.

**What are the risks of being in this study? Is there any way being in this study could be bad for me?**
Based on our research to date, we fully expect that this research will positively impact you. We will be talking about sensitive topics, such as how girls think and feel about their bodies. We do not anticipate that taking part in the sessions will cause you distress. In the unlikely event that this occurs, support strategies will be put in place (e.g., you will be provided with links to additional support services).

**Will it cost me anything to participate in this research study?**
There will be no cost to you for any of the study activities or procedures.

**Will being in this study help me in any way? (Detailed Benefits)**
You will play an integral role in developing a program that helps girls and coaches address body image concerns within sport settings. Feedback from participants who have participated in similar research indicates that these sessions are interesting and enjoyable. Lastly, in recognition of your contribution to this program, you will receive a small monetary compensation for your time (up to $100).

**What happens to the information collected for the research?**
Efforts will be made to limit the use and disclosure of your personal information, including research study records, to people who have a need to review this information. We cannot promise complete secrecy. Organizations that may inspect and copy your information include the IRB and other representatives of this institution.

The researchers collect responses from numerous girls and coaches. Your individual responses will never be shared or discussed beyond the research team. When describing the research results, we will discuss the group as a whole (e.g., overall, a majority of girls enjoyed the **Body Confident Athletes** program). These results will be shared in reports and at conferences, but under no circumstances will participants' details or responses be shared.

[Page Break]

**Information About This Study (page 4 of 4)**

**Additional sharing of your information for mandatory reporting**
If we learn about any current or ongoing child or vulnerable adult abuse or neglect, we may be required or permitted by law or policy to report this information to authorities.

**Whom do I contact if I have questions, concerns, or feedback about my experience?**
This research has been reviewed and approved by an Institutional Review Board (IRB) within the Human Research Protections Program (HRPP). To share feedback privately with the HRPP about your research experience, call the Research Participants’ Advocate Line at 612-625-1650 (toll free: 1-888-224-8636) or go to z.umn.edu/participants. You are encouraged to contact the HRPP if:

- Your questions, concerns, or complaints are not being answered by the research team.
- You cannot reach the research team.
- You want to talk to someone besides the research team.
- You have questions about your rights as a research participant.
- You want to get information or provide input about this research.

**Will I have a chance to provide feedback after the study is over?**
After the study, you might be asked to complete a survey about your experience as a research participant. You do not have to complete the survey if you do not want to. If you do choose to complete the survey, your responses will be anonymous.

If you are not asked to complete a survey after the study is over, but you would like to share feedback, please contact the study team or the Human Research Protection Program (HRPP). See the **“Investigator Team Contact Information”** of this form for study team contact information and **“Whom do I contact if I have questions, concerns, or feedback about my experience?”** of this form for HRPP contact information.

**Will I be compensated for my participation?**
If you agree to take part in this research study, we will provide a small monetary compensation for your time and effort (up to $100).

To save a copy of the information you read on the previous pages: [Click here to download the Information Sheet and save for your records](https://uwe.eu.qualtrics.com/CP/File.php?F=F_8CwiFeQKMJKRH82).

All personal data will be processed in accordance with the following privacy policy: [Click here to download the Privacy Policy and save for your records](https://uwe.eu.qualtrics.com/CP/File.php?F=F_8jPN4D0mIUNzYOO).

**Please indicate if you consent to your and your child's participation:**

- I agree to participate
- I do not agree to participate

**Parents’ Information & Consent Form**


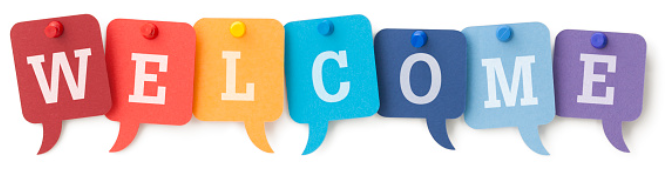


You have received this survey because your child's sports club or school has suggested that your child might like to participate in our research study.

In the first part of the survey you will be provided with information about the research we are conducting on the ***Body Confident Athletes*** program. You will then indicate whether you give consent for your child to participate in the research study.

If you provide consent, we will work with your child's sports club or school to send your child an online survey, where we will also gain their assent for participating.  

You and your child do not have to participate in this research. It is voluntary and you can withdraw at any time!

Completing this survey should take no longer than 10 minutes of your time. Please make sure you complete the whole survey.

[Page Break]

**Information About This Study (page 1 of 4)**

**Title of Research Study:**Developing Embodied Programs for Girls and Coaches

**Investigator Team Contact Information:** Dr Emily Matheson, Dr Courtney Boucher

For questions about research appointments, the research study, research results, or other concerns, contact the study team at:

| **Researcher Name** | Dr Courtney Boucher | **Researcher Name** | Dr Emily Matheson |
| --- | --- | --- | --- |
| **Researcher Affiliation** | Tucker Center for Research on Girls & Women in Sport, University of Minnesota | **Researcher Affiliation** | Centre for Appearance Research, University of the West of England |
| **Email Address** | bouch114@umn.edu | **Email Address** | emily.matheson@uwe.ac.uk |

**Supported By:** Tucker Center for Research on Girls & Women in Sport, Centre for Appearance Research

***Key Information About This Research Study.*** The following is a short summary to help you decide whether or not to be a part of this research study. More detailed information is listed later on in this survey.

**What is research?**
Doctors and researchers are committed to your child’s care and safety. There are important differences between research and treatment plans:

- The goal of research is to learn new things in order to help groups of people in the future. Researchers learn things by following the same plan with a number of participants, so they do not usually make changes to the plan for individual research participants. You, as an individual, may or may not be helped by volunteering for a research study.
- The goal of treatment is to help you get better or to improve your quality of life. Doctors can make changes to your treatment plan as needed.

**Why am I being invited to take part in this research study?**
We are asking you and your child to take part in this research study because you are the parent of a girl aged 11 – 17 years who plays sports. This information form will provide an overview of the research.

**What should I know about being in a research study?**

- Someone will explain this research study to you.
- Whether or not you take part is up to you.
- You can choose not to take part.
- You can agree to take part and later change your mind.
- Your decision will not be held against you.
- You can ask all the questions you want before you decide.

**Why is this research being done?**

Girls often worry about their appearance when playing sport, which can reduce their enjoyment and performance in sport. These worries are worsened by athletic appearance ideals promoted by society, ill-fitting and objectifying uniforms for girls and women, and appearance-related teasing from peers and coaches.

Currently, there is no rigorously tested, effective, or scalable program that is delivered by coaches, and improves girls’ body image and sports enjoyment. We have designed the **Body Confident Athletes** program for girls, which aims to:

- Stop girls from dropping out of sport because of body image concerns.
- Help girls reclaim confidence in their bodies within sport settings.

**How long will the research last?**
We expect that your child’s participation in this research study will last 3 weeks, with the participation in 1-hour weekly sessions. We will aim to schedule these sessions within her regular activities at her sports club or school.

**What will I need to do to participate?**
With your permission and your daughter’s assent, she will participate in a body image program at her sports club or school. The program will include 3 in-person sessions and will be led by her coach. More detailed information about the study procedures can be found under the section **“What happens if I say “Yes, I want to be in this research”?”**.

**Is there any way being in this study could be bad for me or my child?**
Based on our research to date, we fully expect that this research will positively impact your child. We will be talking about sensitive topics, such as how she thinks and feels about her body. However, it is her choice to share as little or as much as she feels comfortable with during the sessions.

**Will being in this study help me or my child in any way?**
Your daughter will play an integral role in testing a program that helps girls and coaches to address body image concerns within sport settings.

**What happens if I do not want to be in this research?**
You and your child may decline to participate and it will not be held against you.

[Page Break]

**Information About This Study (page 2 of 4)**

**Detailed Information About This Research Study.**The following is more detailed information about this study in addition to the information listed on the previous page.

**How many people will be studied?**
We expect about 200 coaches and 2,000 girls will be in this research study over multiple sessions.

**What happens if I say “Yes, I want to be in this research”?**
With your permission and your daughter’s assent, she will participate in a body image program at her sports club or school. The program will include 3 in-person sessions and will be led by her coach. A group of girls and coaches from the club or school will be asked to:

- Complete up to 4 online surveys.
- Participate in a 3 week in-person program that is delivered by the coaches.
- Activities will include group discussions and physical activities, and individual writing and reflection activities aimed at improving girls’ body image.
- Sometimes, we may record the sessions. This is to make sure the coach is delivering the program as planned.
- Before and after your daughter participates in the ***Body Confident Athletes*** program, she will complete an online survey. This survey will ask her questions about herself (e.g., age, the city she lives in) and her well-being. These questionnaires will be anonymized (e.g., we will remove her name or other information that may identify her), and will not be shared or read by anyone, other than the small research team.
- During the program and when answering the online surveys, there are no right or wrong answers.
- What your daughter shares during this research will be confidential. That is, we will not share what she says during the sessions or in the questionnaires. On rare occasions, we may need to share her responses, if someone is worried about her or someone else’s safety.
- In recognition of your daughter’s contribution to this research, she will receive a small monetary compensation for her time. She will receive up to $60 dollars at the end of the study (i.e., after completing the fourth online survey).

**What data do we collect?**
The online surveys include demographic questions and self-report measures that assess your child’s body image, mental health, and well-being. These measures have been validated and peer-reviewed for use among young people and adults. To date, no adverse effects have been reported among participants following exposure to scales of this kind.

**How long do we keep your data for?**
All forms of raw data will be stored for as long as they retain research value. After that time all raw data will be destroyed; however, the project supervisor will retain a computer version of the dataset (e.g., transcriptions, questionnaire responses), which will be stored in password-protected files on a secure server at all times for up to two years after study completion.

Only the core research team will have access to the recordings and questionnaire responses. These data will be shared with co-investigators and partners (e.g., via Microsoft Teams Share Screen), but they will not retain the data.

All data sets will be password protected, with access granted only to the primary researchers on the project. In the event that data needs to be shared with external collaborators (e.g., the research funders), then it will be shared using University of Minnesota approved methods. All collaborators will also agree to the data management terms for this project.

An optional element will be added to collect email addresses to contact participants about future research opportunities. Email addresses will be stored separately from research data and will not be used to identify participant responses.

[Page Break]

**Information About This Study (page 3 of 4)**

**What happens if I say “Yes”, but I change my mind later?**
Choosing not to be in this study or to stop being in this study will not result in any penalty to you or your child or loss of benefit to which you are entitled. At any time, you and your child may decide to withdraw from the study. If you withdraw, no more information will be collected from you or your child. When you indicate that you wish to withdraw, the information already collected will be used in the study unless you specify that you would like your or your child’s data destroyed.

**What are the risks of being in this study? Is there any way being in this study could be bad for me or my child?**
Based on our research to date, we fully expect that this research will positively impact your child. We will be talking about sensitive topics, such as how she thinks and feels about her body. However, it is her choice to share as little or as much as she feels comfortable with during the sessions.

We do not anticipate that taking part in the program will upset your child. In the unlikely event that this occurs, support strategies will be put in place (e.g., she and her coach will discuss her concerns and be provided with links to additional support services).

**Will it cost me anything to participate in this research study?**
There will be no cost to you for any of the study activities or procedures.

**Will being in this study help me or my child in any way? (Detailed Benefits)**
Your daughter will play an integral role in testing a program that helps girls and coaches to address body image concerns within sport settings. Feedback from girls who have participated in similar research, indicate that these sessions are interesting and enjoyable. Lastly, in recognition of your child’s contribution to this study, your daughter will receive a small monetary compensation (up to $60) for her time.

**What happens to the information collected for the research?**
Efforts will be made to limit the use and disclosure of your and your child’s personal information, including research study records, to people who have a need to review this information. We cannot promise complete secrecy. Organizations that may inspect and copy your information include the IRB and other representatives of this institution.

The researchers collect responses from numerous girls and coaches. Your child’s individual responses will never be shared or discussed beyond the research team. When describing the research results, we will discuss the group as a whole (e.g., overall, a majority of girls enjoyed the***Body Confident Athletes*** program). These results will be shared in reports and at conferences, but under no circumstances will participants' details or responses be shared.

[Page Break]

**Information About This Study (page 4 of 4)**

**Additional sharing of your information for mandatory reporting**
If we learn about any current or ongoing child or vulnerable adult abuse or neglect, we may be required or permitted by law or policy to report this information to authorities.

**Whom do I contact if I have questions, concerns, or feedback about my experience?**
This research has been reviewed and approved by an Institutional Review Board (IRB) within the Human Research Protections Program (HRPP). To share feedback privately with the HRPP about your or your child’s research experience, call the Research Participants’ Advocate Line at 612-625-1650 (toll free: 1-888-224-8636) or go to z.umn.edu/participants. You are encouraged to contact the HRPP if:

- Your questions, concerns, or complaints are not being answered by the research team.
- You cannot reach the research team.
- You want to talk to someone besides the research team.
- You have questions about your or your child’s rights as a research participant.
- You want to get information or provide input about this research.

**Will I have a chance to provide feedback after the study is over?**
After the study, you might be asked to complete a survey about your child’s experience as a research participant. You do not have to complete the survey if you do not want to. If you do choose to complete the survey, your responses will be anonymous.

If you are not asked to complete a survey after the study is over, but you would like to share feedback, please contact the study team or the Human Research Protection Program (HRPP). See the **“Investigator Team Contact Information”** of this form for study team contact information and **“Whom do I contact if I have questions, concerns, or feedback about my experience?”** of this form for HRPP contact information.

**Will my daughter be compensated for her participation?**
Your daughter will receive a small monetary compensation for her participation in this study (up to $60). All payments will be given directly to her.

To save a copy of the information you read on the previous pages: [Click here to download the Information Sheet and save for your records](https://uwe.eu.qualtrics.com/CP/File.php?F=F_8CwiFeQKMJKRH82).

All personal data will be processed in accordance with the following privacy policy: [Click here to download the Privacy Policy and save for your records](https://uwe.eu.qualtrics.com/CP/File.php?F=F_8jPN4D0mIUNzYOO).

**Please indicate if you consent to your and your child's participation:**

- I agree to participate
- I do not agree to participate

**Girls’ Informed Assent**


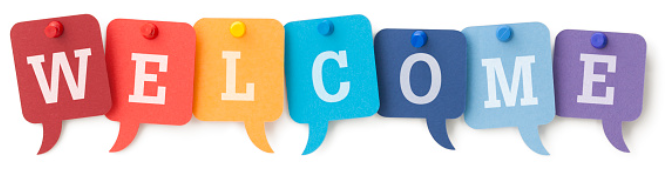


Hello! We are the research team behind the ***Body Confident Athletes*** program.

We are reaching out to you because your parent or guardian thinks you might like to participate in our research.

In the first part of this survey you will learn about the research we are doing on the **Body Confident Athletes**program. Then, you will tell us if you agree to participate - this means that you give 'consent' to participate in research.

If you agree to participate, you will move on to part two, where you will answer questions about your mental health and well-being.

You do not have to participate in this research. It is voluntary and you can stop at any time!

Completing this survey should take no more than 20 minutes.

[Page Break]

**Part One - Research Information**

**Information About This Study (page 1 of 3)**

**Title of Research Study:**Developing Embodied Programs for Girls and Coaches

**Investigator Team Contact Information:**Dr Courtney Boucher, Dr Emily Matheson

**Supported By:**Tucker Center for Research on Girls & Women in Sport, Centre for Appearance Research (CAR)

**What is research?**
Researchers are committed to your care and safety. There are important differences between research and treatment plans:

- The goal of research is to learn new things in order to help groups of kids in the future. Researchers learn things by asking a question, making a plan, and testing it.
- The goal of treatment is to help you get better by using medication, therapy, surgery, or other things that usually make kids feel better. Sometimes treatments help make you feel better or get rid of the condition completely.

**Why am I being asked to take part in this research study?**
A research study is usually done to find a better way to treat people or to understand how things work. You are being asked to take part in this research study because you are a girl aged 11 – 17 years who plays sports. Your parent or guardian has indicated that you might like to participate in our research study. This information form will provide an overview of the research.

**What should I know about being in a research study?**
You do not have to participate in this study if you do not want to. It is completely up to you. We suggest speaking with your parents or guardians if you have any questions about this research project. You can also change your mind about participating. For example, you can start the project and then pull out half way through. If you choose to pull out, no one will be mad.

**Information About This Study (page 2 of 3)**

**Why is this research being done?**

Research shows that girls stop doing activities they enjoy (like playing sports) because they are worried about how they look. We have created a program that helps girls to feel more confident about their bodies when playing sports.

**How long will the research last?**
The ***Body Confident Athletes*** program takes 3 weeks to complete. You will join a 1-hour lesson each week, for 3 weeks. These sessions will be led by your coach at your local sports club or school. We will schedule these lessons with your coach, to fit your regular activities.

**What happens if I say “Yes, I want to be in this research”?**
The ***Body Confident Athletes*** program consists of 3 sessions delivered by your coach. Your teammates and a coach from your sports club or school will be asked to:

- Participate in a 3-week in-person program that is delivered by the coaches.
- Sessions will include group discussions and physical activities, and individual writing and reflection activities.
- Sometimes, we may audio record the sessions. This is to check whether the program was delivered as planned. The recordings will not be shared with anyone other than the research team.
- Before and after you join the sessions, you will complete an online survey with questions about yourself (for example, your age, the city you live in) and your well-being. There will be four surveys in total.
- We will remove your name and other information that could identify you from the survey. Your responses will not be shared or read by anyone, other than the small research team.
- During the sessions and when answering the online surveys, there are no right or wrong answers.
- What you share in this research will be confidential. That is, we will not share what you say during the sessions or in the surveys. On rare occasions, we may need to share your responses if someone is worried about your or someone else’s safety.
- As a thank you, you will receive up to $60 dollars at the end of the study (i.e., after completing the fourth online survey).

**What data do we collect?**
The online surveys ask you questions about yourself, like your age, how long you have played sport, and questions about your mental health and well-being. These questions are frequently used with young people. Young people usually report feeling OK after answering these questions.

**How long do we keep your data for?**
We will keep your answers for as long as we can use them for research. After that time your answers will be deleted. The project supervisor will keep a computer version of your answers for no more than two years after the research ends. Your answers will always be protected by a password on a secure server.

Only the small research team will have access to the recordings and survey responses. When we share girls' survey responses, we will discuss patterns that we see across all girls in the research project. We will not discuss individual girls' responses. This information will be shared with co-investigators and partners (e.g., via Microsoft Teams Share Screen), but they will not keep this information.

All girls' survey responses will be password protected, only the small research team will have access. In case it needs to be shared with external collaborators (e.g., the research funders), then it will be shared using University of Minnesota approved methods. All collaborators of this research will also agree to how the information is managed in this research.

We will ask for your email address for future research opportunities. We will separate email addresses from survey answers and email addresses will not be used to identify participants.

**Information About This Study (page 3 of 3)**

**Is there any way being in this study could be bad for me?**
Based on what we know, we expect that this research will have a positive impact on you. You will be talking about sensitive topics, such as how you think and feel about your body, but you can share as little or as much as you feel comfortable.

We do not think that attending the sessions will upset you. In the unlikely event that this happens, you will be provided with information to support services.

**What happens to the information collected for the research?**
The researchers will share your information only with people who need to review this information. For example, sometimes researchers need to share this information with their university or other people that work in research to make sure the researchers are following the rules. When describing the research results, we will discuss the group as a whole (for example, *a majority of girls enjoyed the****Body Confident Athletes****program*). These results will be shared in reports and at conferences, but individual participants’ details or responses will never be shared.

**What else do I need to know?**
If you participate in this research, the research team will give you up to $60 at the completion of the fourth survey, as a thank you for sharing your time and thoughts with us.

**Who can I talk to?**
For questions about the program sessions, the research, results, or other concerns, contact the research team at:

| **Researcher Name** | Dr Courtney Boucher | **Researcher Name** | Dr Emily Matheson |
| --- | --- | --- | --- |
| **Researcher Affiliation** | Tucker Center for Research on Girls & Women in Sport, University of Minnesota | **Researcher Affiliation** | Centre for Appearance Research, University of the West of England |
| **Email Address** | bouch114@umn.edu | **Email Address** | emily.matheson@uwe.ac.uk |

This research has been reviewed and approved by an Institutional Review Board (IRB) within the Human Research Protections Program (HRPP). To share feedback privately with the HRPP about your research experience, call the Research Participants’ Advocate Line at 612-625-1650 (toll free: 1-888-224-8636) or go to z.umn.edu/participants. You are encouraged to contact the HRPP if:

- Your questions, concerns, or complaints are not being answered by the research team.
- You cannot reach the research team.
- You want to talk to someone besides the research team.
- You have questions about your rights as a research participant.
- You want to get information or provide input about this research.

To save a copy of the information you read on the previous pages: [Click here to download the Information Sheet and save for your records](https://uwe.eu.qualtrics.com/CP/File.php?F=F_8CwiFeQKMJKRH82).

All personal data will be processed in accordance with the following privacy policy: [Click here to download the Privacy Policy and save for your records](https://uwe.eu.qualtrics.com/CP/File.php?F=F_8jPN4D0mIUNzYOO).

**Please indicate if you consent to your and your child's participation:**

- I agree to participate
- I do not agree to participate

**Sample of Girls’ Questionnaire administered at Baseline (T1), Post-Intervention (T2), 1-month (T3) and 3-Month (T4) Follow-Up**

Thank you for agreeing to participate in this research.

 You will now answer some **questions about yourself**and **your well-being**.
Please press the yellow arrow button to start.

[Page Break]

**Part Two - Demographic Questions**

Please create your participant ID. You will need this ID again in the next surveys. This helps us match your responses from different surveys without keeping your personal information.

We suggest you write your ID down and keep it in a safe place. You can also ask a parent or trusted adult to support you with creating this ID.

Your participant ID is a series of 8 numbers and letters, as follows:

1. The**first two** letters of your **first name** (e.g., **Sa**rah)
2. Your **birth date** (e.g., **17**th of October, 2005)
3. The **first**letter of your**birth month** (e.g., **J**, **F**, **M**, etc.)
4. The **first two** letters of your **mother's first name** (e.g., **Ma**ry)
5. The **first** letter of the **state**where you live (e.g., **T**exas)

For example, **Sa**rah is born on **17**th **O**ctober, her mother's name is **Ma**ry, and she lives in **T**exas. Her code would be: **Sa17OMaT**

[Page Break]

**How old are you?**

- 10 or younger (1)
- 11 (2)
- 12 (3)
- 13 (4)
- 14 (5)
- 15 (6)
- 16 (7)
- 17 (8)
- 18 or older (9)

**Which gender do you identify with?**

- Male (1)
- Female (2)
- Non-binary/third gender (3)
- Prefer to self-describe: (4) __________________________________________________

**How would you best describe yourself?**

- Asian (1)
- Black or African American (2)
- Hispanic, Latino/a, or Spanish origin (3)
- Middle Eastern or North African (4)
- Native American or Alaska Native (5)
- Native Hawaiian or Other Pacific Islander (6)
- White (7)
- Multiracial or Biracial (8)
- Prefer not to say (9)
- Prefer to self-describe: (10) __________________________________________________

**Do you identify as someone with a physical disability?**

- Yes (1)
- No (2)

**What city do you live in?**

________________________________________________________________

**What is the name of the sports club or school where you will participate in the *Body Confident Athletes* program?**

________________________________________________________________

SO Length **How long have you been playing sport?**

- Less than 1 month (1)
- 1-6 months (2)
- 6-12 months (3)
- More than 1 year (4)
- More than 3 years (5)
- More than 5 years (6)

[Page Break]

**Please indicate how often you agree with the following statements ranging from "never" (0) to "always" (4).**

|  | **0 - Never** (1) | **1 - Rarely (2)** | **2 - Sometimes (3)** | **3 - Often (4)** | **4 - Always (5)** |
| --- | --- | --- | --- | --- | --- |
| 1. I like what I look like in pictures (1) |  |  |  |  |  |
| 2. I'm proud of my body (2) |  |  |  |  |  |
| 3. I am preoccupied with trying to change my body weight (3) |  |  |  |  |  |
| 4. I like what I see when I look in the mirror (4) |  |  |  |  |  |
| 5. There are lots of things I'd change about my looks if I could (5) |  |  |  |  |  |

[Page Break]

**Please indicate how often you agree with the following statements ranging from "never" (0) to "always" (4).**

|  | **0 - Never (1)** | **1 - Rarely (2)** | **2 - Sometimes (3)** | **3 - Often (4)** | **4 - Always (5)** |
| --- | --- | --- | --- | --- | --- |
| 6. I am satisfied with my weight (1) |  |  |  |  |  |
| 7. I wish I looked better (2) |  |  |  |  |  |
| 8. I really like what I weigh (3) |  |  |  |  |  |
| 9. I wish I looked like someone else (4) |  |  |  |  |  |
| 10. My looks upset me (5) |  |  |  |  |  |
| 11. I'm pretty happy about the way I look (6) |  |  |  |  |  |

**Please indicate how often you agree with the following statements ranging from "never" (0) to "always" (4).**

|  | **0 - Never (1)** | **1 - Rarely (2)** | **2 - Sometimes (3)** | **3 - Often (4)** | **4 - Always (5)** |
| --- | --- | --- | --- | --- | --- |
| 12. I feel I weigh the right amount for my height (1) |  |  |  |  |  |
| 13. I feel ashamed of how I look (2) |  |  |  |  |  |
| 14. Weighing myself depresses me (3) |  |  |  |  |  |
| 15. My weight makes me unhappy (4) |  |  |  |  |  |
| 16. I worry about the way I look (5) |  |  |  |  |  |
| 17. I think I have a good body (6) |  |  |  |  |  |
| 18. I'm looking as nice as I'd like to (7) |  |  |  |  |  |

[Page Break]

**An athlete may enjoy several things about sports. Enjoyment can be thought of as experiences or events that lead to positive feelings of pleasure, liking, and fun.

 Please think about your entire experience in sport: the competitions, practices, time away from your sport environment, and your experiences with other people involved in your sport participation.

 Think not only about your present experience, but your experience in sports overall, then answer the following questions. There are no right or wrong answers, so please respond honestly. Please indicate your answer to the bolded statement by clicking on the number that follows each item.**

***During the times when I most enjoy sport, I usually experience that enjoyment from…***

|  | **1 - Strongly Disagree (1)** | **2 - Disagree (2)** | **3 - Neither Agree Nor Disagree (3)** | **4 - Agree (4)** | **5 - Strongly Agree (5)** |
| --- | --- | --- | --- | --- | --- |
| 1. Playing up to my potential (1) |  |  |  |  |  |
| 2. Working hard in practice (2) |  |  |  |  |  |
| 3. Improvement of my performance based on my ability to outperform others (3) |  |  |  |  |  |
| 4. Being with the friends on my team (4) |  |  |  |  |  |
| 5. Doing skills other kids my age cannot do (5) |  |  |  |  |  |
| 6. The feeling of team spirit and togetherness I feel from being on a team (6) |  |  |  |  |  |
| 7. Getting support and encouragement from my teammates (7) |  |  |  |  |  |

[Page Break]

***During the times when I most enjoy sport, I usually experience that enjoyment from…***

|  | **1 - Strongly Disagree (1)** | **2 - Disagree (2)** | **3 - Neither Agree Nor Disagree (3)** | **4 - Agree (4)** | **5 - Strongly Agree (5)** |
| --- | --- | --- | --- | --- | --- |
| 8. Participating in a close game, meet, or competition (1) |  |  |  |  |  |
| 9. Participating in and finishing a difficult practice (2) |  |  |  |  |  |
| 10. Making new friends in my sport (3) |  |  |  |  |  |
| 11. Doing things with my teammates away from practice or competition (4) |  |  |  |  |  |
| 12. Being known by others for being an athlete (5) |  |  |  |  |  |
| 13. Playing hard during competition (6) |  |  |  |  |  |
| 14. Improvement of performance based on how I’ve done in the past (7) |  |  |  |  |  |
| 15. For this item, please select 'Agree' (8) |  |  |  |  |  |

[Page Break]

***During the times when I most enjoy sport, I usually experience that enjoyment from…***

|  | **1 - Strongly Disagree (1)** | **2 - Disagree (2)** | **3 - Neither Agree Nor Disagree (3)** | **4 - Agree (4)** | **5 - Strongly Agree (5)** |
| --- | --- | --- | --- | --- | --- |
| 16. Hearing the crowd cheer during a close game, match, or race (1) |  |  |  |  |  |
| 17. Showing that I am better than others who play my sport (2) |  |  |  |  |  |
| 18. Getting encouragement from my parent(s) (3) |  |  |  |  |  |
| 19. Being better in my sport than other athletes my age or in my league (4) |  |  |  |  |  |
| 20. Being recognized by others because I participate in sport (5) |  |  |  |  |  |
| 21. Feeling exhausted after a practice or competition (6) |  |  |  |  |  |
| 22. Playing well compared to how I’ve played in the past (7) |  |  |  |  |  |

[Page Break]

***During the times when I most enjoy sport, I usually experience that enjoyment from…***

|  | **1 - Strongly Disagree (1)** | **2 - Disagree (2)** | **3 - Neither Agree Nor Disagree (3)** | **4 - Agree (4)** | **5 - Strongly Agree (5)** |
| --- | --- | --- | --- | --- | --- |
| 23. The thrill of competition (1) |  |  |  |  |  |
| 24. Getting support from my parent(s) for playing my sport (2) |  |  |  |  |  |
| 25. The excitement of competition (3) |  |  |  |  |  |
| 26. Having my parent(s) watch me compete (4) |  |  |  |  |  |
| 27. Giving a lot of effort in practice or competition (5) |  |  |  |  |  |
| 28. Achieving personal goals I set for myself based on my own performances (6) |  |  |  |  |  |
| 29. Having my parent(s) pleased with my performance no matter what (7) |  |  |  |  |  |

[Page Break]

**Please indicate whether the question is true about you never, rarely, sometimes, often, or always.**

|  | **1 - Never (1)** | **2 - Rarely (2)** | **3 - Sometimes (3)** | **4 - Often (4)** | **5 - Always (5)** |
| --- | --- | --- | --- | --- | --- |
| 1. I respect my body (1) |  |  |  |  |  |
| 2. I feel good about my body (2) |  |  |  |  |  |
| 3. I feel that my body has at least some good qualities (3) |  |  |  |  |  |
| 4. I take a positive attitude towards my body (4) |  |  |  |  |  |
| 5. I am attentive to my body's needs (5) |  |  |  |  |  |

[Page Break]

**Please indicate whether the question is true about you never, rarely, sometimes, often, or always.**

|  | **1 - Never (1)** | **2 - Rarely (2)** | **3 - Sometimes (3)** | **4 - Often (4)** | **5 - Always (5)** |
| --- | --- | --- | --- | --- | --- |
| 6. I feel love for my body (6) |  |  |  |  |  |
| 7. I appreciate the different and unique characteristics of my body (7) |  |  |  |  |  |
| 8. My behavior reveals my positive attitude toward my body; for example, I hold my head high and smile (8) |  |  |  |  |  |
| 9. I am comfortable in my body (9) |  |  |  |  |  |
| 10. I feel like I am beautiful even if I am different from media images of attractive people (e.g., models, actresses/actors) (10) |  |  |  |  |  |

[Page Break]

**Please choose the number from the following that best describes how you feel about each of the statements listed below. Indicate your response by selecting a number beside each statement.**

**That is: “1” if you Strongly Disagree; “2” if you Somewhat Disagree; “3” if you Neither Agree Nor Disagree; “4” if you Somewhat Agree; or “5” if you Strongly Agree**

|  | **1 - Strongly Disagree (1)** | **2 - Somewhat Disagree (2)** | **3 - Neither Agree Nor Disagree (3)** | **4 - Somewhat Agree (4)** | **5 - Strongly Agree (5)** |
| --- | --- | --- | --- | --- | --- |
| 1. I care more about how my body feels than about how it looks (4) |  |  |  |  |  |
| 2. I focus more on what my body can do than on its appearance (5) |  |  |  |  |  |
| 3. I spend a lot of time/energy/money engaging in activities that I hope make me fit with cultural ideals of beauty (e.g., exercise, clothing, make-up, hair, plastic surgery, skin bleaching) (7) |  |  |  |  |  |
| 4. I constantly think about whether my body is considered attractive or beautiful to others (10) |  |  |  |  |  |

[Page Break]

**Please choose the number from the following that best describes how you feel about each of the statements listed below. Indicate your response by selecting a number beside each statement.**

**That is: “1” if you Strongly Disagree; “2” if you Somewhat Disagree; “3” if you Neither Agree Nor Disagree; “4” if you Somewhat Agree; or “5” if you Strongly Agree**

|  | **1 - Strongly Disagree (1)** | **2 - Somewhat Disagree (2)** | **3 - Neither Agree Nor Disagree (3)** | **4 - Somewhat Agree (4)** | **5 - Strongly Agree (5)** |
| --- | --- | --- | --- | --- | --- |
| 1. I engage in potentially harmful or painful behaviors (e.g., disordered eating, bingeing, purging, denying physical needs, skin cutting, burning, drug use, excessive alcohol consumption) (7) |  |  |  |  |  |
| 2. I have an eating disorder (8) |  |  |  |  |  |
| 3. I take good care of, and am respectful of, my body (10) |  |  |  |  |  |
| 4. For this item, please select 'Somewhat Disagree' (11) |  |  |  |  |  |
| 5. I ignore the signs my body sends me (e.g., of hunger, stress, fatigue, illness/injury) (6) |  |  |  |  |  |
| 6. I am aware of my needs (4) |  |  |  |  |  |
| 7. It is hard for me to read/identify my feelings (3) |  |  |  |  |  |
| 8. I make sure I listen to my body and its needs (e.g., rest when I am tired, eat when hungry, leave when I feel unsafe, relax when stressed) (2) |  |  |  |  |  |

[Page Break]

**The last question asks about how you feel. Indicate how often you currently experience these feelings by selecting a number beside each statement.

 That is: “1” for Very Slightly Or Not At All; “2” for A Little; “3” for Moderately; “4” for Quite A Bit; or “5” for Extremely**

|  | **1 - Very Slightly Or Not At All (1)** | **2 - A Little (2)** | **3 - Moderately (3)** | **4 - Quite A Bit (4)** | **5 - Extremely (5)** |
| --- | --- | --- | --- | --- | --- |
| 1. Sad (15) |  |  |  |  |  |
| 2. Happy (3) |  |  |  |  |  |
| 3. Scared (14) |  |  |  |  |  |
| 4. Miserable (11) |  |  |  |  |  |
| 5. Cheerful (2) |  |  |  |  |  |
| 6. Proud (5) |  |  |  |  |  |
| 7. Afraid (13) |  |  |  |  |  |
| 8. Joyful (1) |  |  |  |  |  |
| 9. Mad (12) |  |  |  |  |  |
| 10. Lively (4) |  |  |  |  |  |

[Page Break]


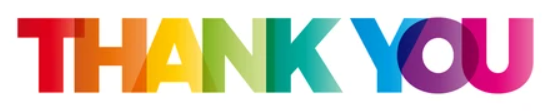


Thank you for reading this information and for answering the survey. Your coach will tell you when you will join the sessions of the ***Body Confident Athletes*** program.

If you have any questions or concerns please contact our research team: Dr Emily Matheson ([emily.matheson@uwe.ac.uk](http://emily.matheson@uwe.ac.uk/)) or Dr Courtney Boucher ([bouch114@umn.edu](http://nmlavoi@umn.edu/)).
